# Water Adsorption Properties of Boron Carbonitride Monolayers: Effects of Substitution Patterns and Alumina Support

**DOI:** 10.1021/acsomega.5c11128

**Published:** 2026-02-04

**Authors:** Marcos Rivera-Almazo, Bartolomeo Civalleri, Lorenzo Maschio

**Affiliations:** Dipartimento di Chimica, 9314 Università di Torino, Via P. Giuria 5, 10125 Torino, Italy

## Abstract

Graphene and monolayered h-BN materials are promising 2D systems whose properties are appealing for diverse applications, including their use as coatings. Most interestingly, they have the same structure and can be combined in almost infinite ways, giving rise to intermediate systems, known as boron carbonitrides (BCN), with tunable or even enhanced features with respect to the pristine materials. In this work, we study through theoretical quantum-mechanical (DFT) calculations how different BCN systems interact with the H_2_O molecule, which results in a stronger interaction in comparison to the pristine cases. We also consider the support of single-layer BCN on top of the (0001) α-Al_2_O_3_ surface. The formation of the heterostructure determines changes to the on-top 2D systems that are expected to modify the interaction with water.

## Graphical abstract

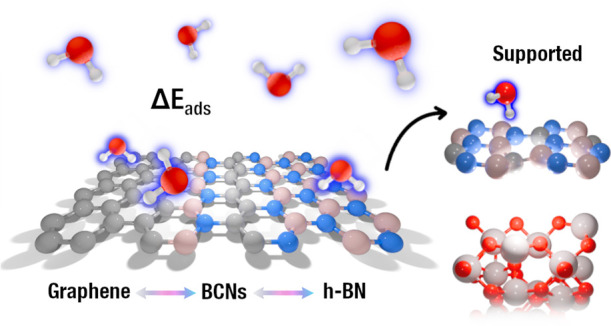

## Introduction

Graphene-based materials continue to be among the most interesting and reviewed ones.^1–6^ Different structural and composition modifications have been explored for pristine graphene (pG), looking to enhance its thermal or mechanical properties, or to open a small gap in its semimetallic band structure.^7–12^ Similarly, the pristine hexagonal-boron nitride (h-BN) monolayer (pBN) has appealing properties,^13^ having instead a significant gap value (6 eV^14^) which can be excessive for certain applications. At the intersection of both systems, boron carbonitrides, BCN (also found in the literature as BN-doped or substituted graphene, but here referenced as BCN to avoid indicating which system is being modified), have been the subject of interest given they are expected to display properties intermediate to their pristine counterparts.^15–17^ Investigations on their nature have been performed using both experimental^18,19^ and theoretical^20–30^ techniques, in general showing them as a promising family of systems.

Regarding adsorption phenomena on top of this monolayer system, that with the H_2_O molecule can be considered one of the most interesting ones given its highly probable presence under many working conditions. Previous theoretical works have explored at diverse degrees of accuracy how pG and pBN interact with water.^31–36^ While density functional theory (DFT) methodologies augmented with a dispersion correction (DFT-D) overestimate the interaction energy with respect to wave function correlated methods-the predicted value is overall below 3.5 kcal/mol^31,32,34,37^ -the relative ordering of such interaction energies is correctly recovered, with a slightly more favorable interaction on pBN than pG. Instead, theoretical methods differ in the preferred interaction site and geometry, with the latter being usually predicted to be H atom-oriented.^31,32,34,37^

The water-adsorption behavior of this material, which is in general associated with the freestanding cases, might change when the monolayer is supported on top of a surface. This is not so uncommon either under certain production conditions or when the monolayer is intended to be used for coating. Indeed, graphene and graphene-based coatings have been tested to improve different types of surfaces.^8,38^ For instance, its support on top of α-Al_2_O_3_, hereafter referred to as alumina, has been considered both as a reinforcing coating^39,40^ and for its usage in electronic devices.^41–44^ Most of those studies consider the α-Al_2_O_3_(0001) surface, for the simple structure of this facet, its stability, and because of the small mismatch it has with the lattice parameters of pG.^39,41–43,45^

The present work explores (i) the different effects that selected substitution patterns have on the electronic, spectral, and water adsorption properties of BCN systems and (ii) how they change when such systems are supported on top of alumina. The comparison here presented for graphene, h-BN and BCN, in both their freestanding and alumina-supported states, provides relevant and novel observations for the development and characterization of BCN materials where the adsorption of water can play a key role.

## Methodology

We carried out periodic electronic structure calculations using the CRYSTAL23 code,^46^ which is based on a linear combination of atomic orbitals (LCAO) expressed in terms of Gaussian-type basis functions. The Perdew–Burke–Ernzerhof (PBE)^47^ generalized gradient approximation (GGA) functional was used for all calculations. Such choice was made based on several considerations: (i) in a previous work,^26^ we have seen that PBE performs as well as the Becke-3-parameters, Lee–Yang–Parr (B3LYP) hybrid functional in vibrational frequencies of BN and graphene, and even slightly better in comparison with experimental references; (ii) such choice allows direct comparison with analogous calculations performed with plane waves; (iii) interaction energies are almost purely dispersive here and thus captured by the additional dispersion correction rather than by the functional; and (iv) the systems themselves and the properties observed are not very sensitive to functionals, and PBE is a convenient choice being slightly less costly than a hybrid functional.

The dispersion correction D3 with Becke–Johnson damping^48–50^ was included in order to have a better description of the noncovalent interactions,^51^ which are expected to be relevant in both H_2_O-monolayer and monolayer-alumina cases. For the BCN systems, system-specific atomic basis sets of triple-ζ (TZ) quality were chosen. Starting from the def2-TZVP molecular ones,^52–54^ the outermost exponents were reoptimized using the basis-direct inversion in the iterative subspace (B-DIIS) algorithm available in CRYSTAL23.^55^ Namely, for B and N basis pBN was used as the reference system for the optimization, while for C basis graphite was employed. The POB-TZVP basis^56^ and def2-TZVP were used for the alumina slab and the water molecules, respectively. 2D periodic conditions (on the *xy* plane) are considered, for which the consideration of vacuum in the nonperiodic direction (*z*-axis) is not required. Further details on the computational parameters and unit cells employed can be found in the SI (CRYSTAL Calculations Settings).

For the interaction with water, the basis set superposition error (BSSE) was estimated by using both the geometrical counterpoise (gCP) method (def2-TZVP/PBE parametrization in CRYSTAL)^57^ and, for one H_2_O@Graphene case, the Boys–Bernardi approach (BB).^58^ This resulted in interaction energy changes between 7 and 17 meV using gCP and of 55 meV using BB. As we considered these values to be small, probably due to the large size of the considered basis sets, we do not include such corrections in this work.

Initial geometries for water molecules in interaction with the freestanding layers were guessed by pairing the electrostatic potential (EP) maps of the surface and the adsorbate. We favored the interaction of water’s hydrogen atoms (with positive EP) with negative EP regions of the layers, as that interaction has been reported to be the strongest in pG.^31,32^ Molecules were initially positioned parallel to the layer, with the exception of a two-legged configuration that in each case was intended to interact simultaneously with two negative (or close to zero) EP regions; this choice is based on the most stable complex reported in the literature between water and pG.^31^

As a model of the (0001) α-Al_2_O_3_ surface, an Al-terminated slab with 18 atomic layers (30 atoms) was selected, with 12 Al and 18 O atoms in the unit cell. A double-sided model with preserved inversion symmetry was chosen to avoid the appearance of a dipole moment along the nonperiodic direction. After an assessment of the EP map of this system and those of the freestanding monolayers, initial configurations for each Monolayer@Surface were proposed (SI).

Interaction energies, *E*_int_, were calculated with the following expression:

$$E_{\mathrm{int}}=\frac{E_{\mathrm{Host}+\mathrm{Guest}}-(h\times E_{\mathrm{Host}}+g\times E_{\mathrm{Guest}})}{n}$$ 1

with *E*_Host+Guest_ being the energy of the optimized complex, which in this study can be H_2_O@Monolayer or Monolayer@Alumina. *E*_Host_ and *E*_Guest_ are the energies of the individually optimized complex parts. For H_2_O@Monolayer (and H_2_O@supported monolayer), *g* = 1 as we consider only a single H_2_O molecule per unit cell. Other than this, *h* and *g* are selected to scale for the number of unit cells used for the Host+Guest complexes; we used at least 3 × 3 supercells (relative to the pG one) to avoid the interaction between periodic H_2_O molecules. Larger 4 × 4 supercells were required when the base system (some BCNs and the alumina-surface model) was only possible to model with 2 × 2 supercells. *n* takes into account the number of times the calculated interaction is present; for H_2_O@Monolayer *n* = 1, while for Monolayer@Alumina *n* = 2 given our double-sided model. No energy decomposition analysis (EDA) of the dispersion contribution to the interaction energy was performed.

Infrared (IR) and Raman spectra were calculated using the default procedure in CRYSTAL, which allows a fully analytical evaluation of the intensities using a couple-perturbed approach.^59–62^

Images included were produced by using the following tools: CRYSTALpytools (IR and Raman spectra),^63^ CRYSPLOT (electron density difference plots, see SI),^64^ VMD (EP maps),^65^ and Beautiful Atoms (atomic structures).^66^

## Results

### IR and Raman Spectra

We validated our methodology by starting from calculations for both pG and pBN systems. For both systems, the cell parameter, band gap, and *E*_2g_ Raman mode associated frequency align with those previously obtained by DFT calculations,^26^ as well as with some of the experimental counterparts (Table 1). After this, we proceeded to perform calculations for the freestanding pristine and BCN systems, obtaining the spectra reported in Figure 1. The doping patterns here considered span a broad range, including BN-pair ratios of 25, 50, and 75%. We confirm the previously estimated Raman spectra for these systems, with the exception of the alt1 case (Figure 2e), an alternative pattern to those previously reported in ref 26. That study also shows that with an appropriate mixture, the simulated spectra reproduce the experimental profile, helping formulate a sensible hypothesis about the corresponding mix of patterns in the material. We also report here the corresponding IR spectra, which in general show complementary information to the one present in the Raman data, given the appearance of additional peaks or the exchange in intensity of others (e.g., for the zz1 pattern).

**Table 1:** Calculated Properties for the Pristine Systems^a^

	*a* (Å)	*E*_2g_ (cm^–1^)	gap (eV)
pG	2.466 (2.46 ^67^)	1571 (1582 ^67^)	0*^b^(0 ^68^)
pBN	2.509 (2.504 ^69^)	1347 (1369 ^70^)	4.67 (6.1 ^14^)

a Experimental values are reported in parentheses.

b The conducting state for graphene is recovered using a Smear parameter of 0.0001.

**Figure 1:**
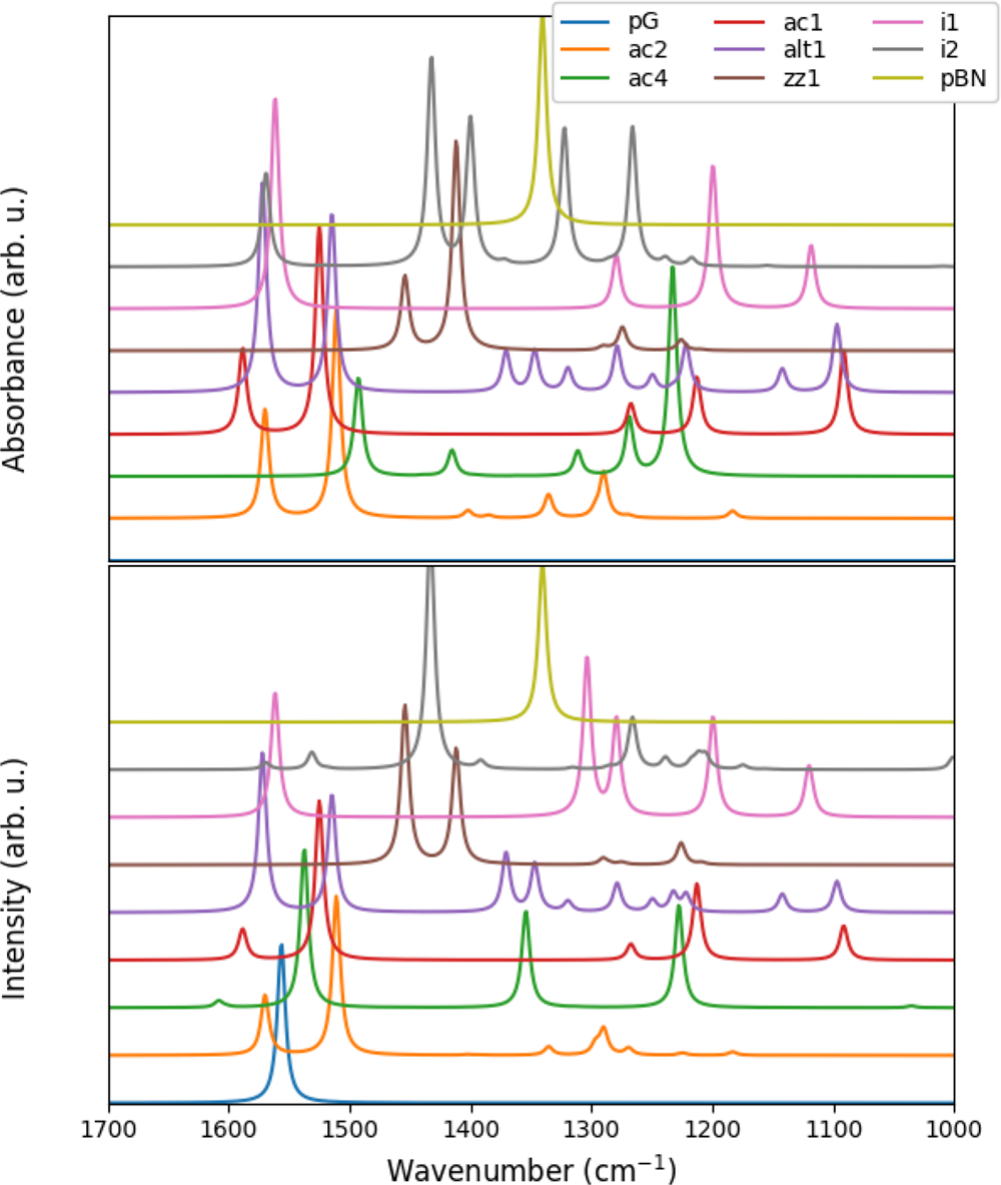
IR (top) and Raman (bottom) spectra obtained for the different BCN patterns.

**Figure 2:**
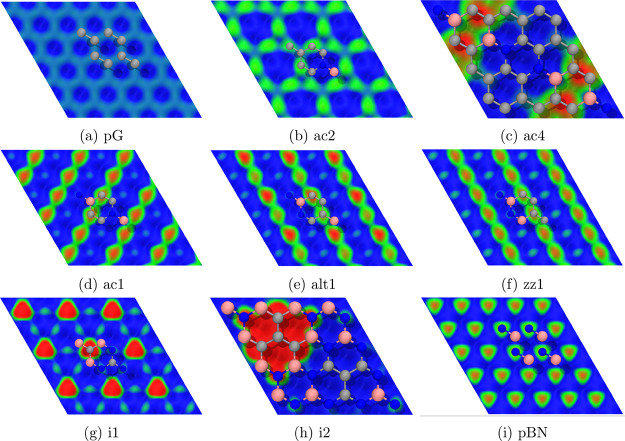
EP maps on top of the freestanding pristine (a, i) and BCN (b–h) monolayers, ρ_cut_ = 0.003, on the [−0.01, 0.01] EP range (red-green-blue color scale). A fragment of each system is shown on top of the ρ surface as a reference. A larger format is available in the SI. Atoms color code: B, pink; C, gray; and N, blue.

For the specific BCN patterns considered, we observe in general a displacement of the G band signal (around 1580 cm^–1^) to lower wavenumbers as the BN percentage increases in both Raman and IR. In all of the studied BCN systems, this mode is also IR-active, in contrast to pG. For alt1, we predict two Raman (and IR) active signals of similar intensity around the pG one, which is not seen for any of the other patterns. The i1 pattern does not follow the displacement trend for this band, with a signal very similar to that observed for pG. The signals in the 1450–1400 range are characteristic of the zz1 and i2 patterns, with a dominant peak in the Raman spectra for i2 and one in the IR for zz1. At wavenumbers lower than 1400, farther than the characteristic peak for pBN, characteristic patterns can be distinguished for the studied BCNs, with both IR and Raman providing complementary information for a more robust identification. These results contribute to the previously available information, helping in identifying a given type of doping pattern or the presence of mixtures, and providing a reference for experimental characterization.

### Water on Freestanding BCN Monolayers

EP maps were obtained for each pristine and BCN system (Figure 2). While for pG there are practically no negative EP regions and for BN these are found on top of the N atoms, we observe that in BCN these negative regions are only found on top of B-atom-bonded C atoms, with the exception of the i2 pattern, where N atoms surrounding the isolated C area extend this negative EP zone. This marks a clear difference in how BCN systems will interact with water or the supporting surface, with B-atom-bonded C atoms playing an important role.

Given the EP maps, different initial H_2_O molecule geometries were proposed, considering the positive EP value in its H atoms and the expected interaction with the B-atom-bonded C atom regions of the BCN layer. After full optimization of 33 initial geometries, 26 unique complexes were recovered in terms of energy and geometry. The range of values for the main characterizing properties of these systems (molecule-layer distance, interaction energy, gap) can be observed in Figure 3 and Table 2; these values are not much spread out for a given BCN, with the exception of systems ac4 and i2, where O-atom-oriented cases were also considered, resulting in those cases having the weakest interaction with the layer (their respective G1 pair geometry; see Table 2), as previously reported for the pristine systems.^31,32,34^ Without considering such cases, we can observe that *E*_int_ does not change considerably for a given monolayer, while the molecule-layer distance has a larger, while still small, variation. Band-gap values are not affected by the molecule adsorption in any of the systems. These results show that the interaction with H_2_O is stronger for the BCN monolayers than for the pristine ones. In particular, the interaction is considerably stronger in the i1 structure, which leads to a distortion of the planar arrangement of the atoms and a shorter molecule-layer distance, as displayed in Figure 4. For comparison, we show the other two structures, ac2 and alt1, which do not show evident distortions. Alt1, in particular, exhibits the second-highest average interaction with water after i1, as seen in Figure 3. Values of *d*_mol–layer_ were calculated as the *z*-coordinate difference between the highest atom of the monolayer and the lowest atom of the molecule. As observed in the EP maps, the B-atom-bonded C atom in i1, which interacts with H_2_O, is associated with a negative and localized EP value, for which the electrostatic contribution of the interaction is higher in comparison to the other cases explored. The resulting force, perpendicular to the monolayer plane, induces the observed out-of-plane distortion. In contrast, the more extended negative EP regions in ac4 and i2 have a repulsive effect with the H_2_O’s oxygen, for which *E*_int_ is predicted to be lower than in i1.

**Table 2:** Interaction Energy, *E*_int_, Band Gap, and Molecule-Layer Distance, *d*_mol‑layer_, Values Obtained for Each of the 26 Studied H_2_O@Monolayer Pairs^a^

layer	pair geometry	*E*_int_ (meV)	gap (eV)	*d*_mol‑layer_ (Å)
pG	G1	162.75	0.0147	2.6036
	G2	167.70	0.0098	2.8332
	G3	190.20	0.0130	2.4913
	S	132.72	1.9656^b^	2.3632
pBN	G1	188.55	4.6440	2.6981
	G2	192.76	4.6440	2.2366
	G3	193.05	4.6445	2.7106
	G4	196.32	4.6408	2.2921
	G5	199.71	4.6417	2.2859
	S	252.21	4.3783	2.4585
ac1	G1	273.93	1.9301	2.4875
	G2	276.43	1.9279	2.5064
ac2	G1	244.70	1.0336	2.5559
	G2	244.89	1.0363	2.4780
	G3	245.65	1.0373	2.5248
alt1	G1	257.15	2.0352	2.2704
	G2	273.63	2.0343	2.3820
	G3	279.23	2.0349	2.3368
zz1	G1	238.21	1.6413	2.3437
	G2	270.24	1.6460	2.4633
i1	G1	455.32	2.8945	2.1578
	G2	460.75	2.8934	1.9234
	S	213.89	1.4783	2.4728
ac4	G1	158.88	0.0571	2.8861
	G2	267.46	0.0444	2.4455
	G3	298.19	0.0552	2.4318
i2	G1	137.59	1.5691	2.7288
	G2	204.60	1.5722	2.7398
	G3	382.23	1.6362	2.0681

a Pair geometry labels are assigned in an increasing order of *E*_int_. The S label indicates the values for alumina-supported geometries reported in the plots.

b As observed for pG, the usage of smearing (0.0001) recovers the conducting state in this case.

**Figure 3:**
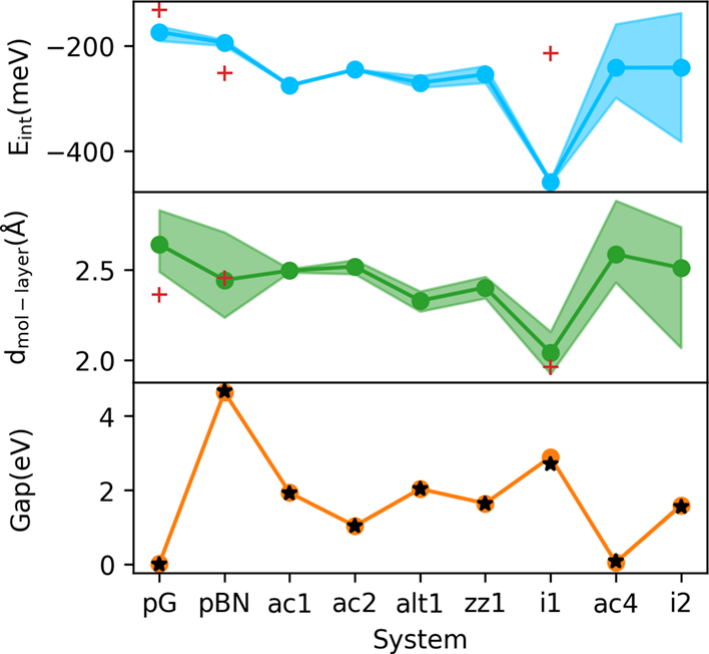
Interaction energy, *E*_int_, molecule-layer distance, *d*_mol–layer_, and band gap obtained for H_2_O@Monolayer pairs. The colored area covers the range of calculated values, while dots indicate the average. Black stars correspond to the band-gap value of the freestanding monolayers. Red crosses indicate values for the H_2_O@Alumina-supported monolayer.

**Figure 4:**
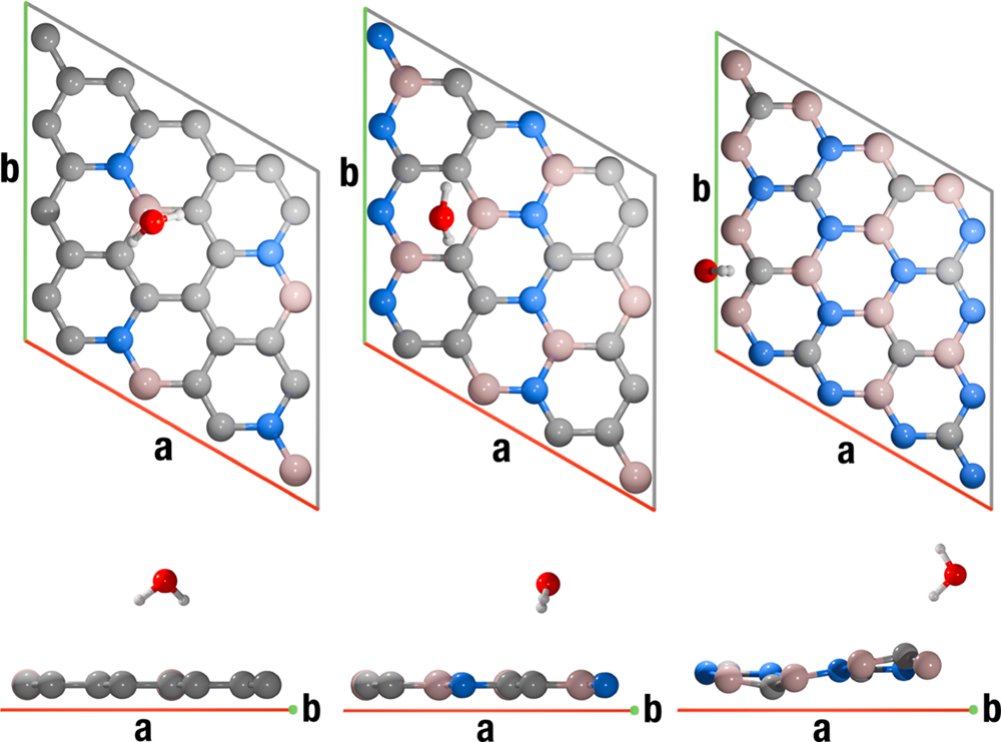
Top (*z*-axis) and lateral (*b*-axis) views of one unit cell for the Water@Monolayer BCN ac2 (left), alt1 (center), and i1 (right) structures. Atom color code: H, white; B, pink; C, gray; N, blue; and O, red.

### BCN Supported on α-Alumina

To simulate an alumina substrate supporting the BCN layer, an 18-layer-thick slab model (with a thickness of 12.49 Å after geometry optimization) was selected for the surface of α-Al_2_O_3_. Convergence with the slab thickness was checked on a surface formation energy test, which we report in the Supporting Information (Figure S26 in the Supporting Information); such convergence is necessary to ensure a correct description of both the outer/surface-like and inner/bulk-like electron densities and consequently enable a better description of the slab–monolayer interaction. Also, the number of layers and thickness of the slab are consistent with previous studies,^41,42^ while they do not justify such a selection. The calculated EP map (Figure S28 in the Supporting Information) showed that the terminating layer Al atoms have positive EP regions; we considered this information to propose an initial geometry for the supported layer, aligning this region with the localized negative EP regions from the freestanding layers.

The results for the optimized Monolayer@Alumina are condensed in Table 3 and Figure 5 for the 9 different structure patterns of the BCN monolayer. Lattice mismatching values (Table S26 in the Supporting Information) indicate that the supported monolayers are the subject of strain given the modeled lateral matching, which increases with the B–N pairs percentage in the system. Different interaction energies were obtained, spanning 2 orders of magnitude. Given the converged surface formation energy, the selected slab thickness, and the opposite orientations of the two monolayers, their mutual influence on adsorption is expected to be small and the observed trends should hold for thicker slabs. *E*_int_ values estimated for the BCN cases are larger than for the pristine ones, with i2 and ac4 having the most relevant adhesion energy. Band-gap values are largely preserved with respect to the freestanding cases, with the exception of the ac1 and i1 systems. Interestingly, both changes decrease the predicted gap, and for ac1, it becomes close to 0. Even more interestingly, the high-interaction layers (i2 and ac4) undergo little variation in the gap. Some of the adsorbed monolayers present distortions from their original planarity, as can be verified from their *z*-coordinate range, Δ*z*_layer_, reported in Table 3. Large Δ*z*_layer_ values, such as those obtained for pBN, alt1, ac4, i1, and i2, indicate large deviation from the original planar geometry of the monolayers after their support. Figure 6 shows graphically how these distortions are presented in supported pG, i1, and pBN.

**Table 3:** Interaction Energy, *E*_int_, Band Gap, Layer–Support Distance, *d*_layer–supp_, and *z*-Range of the Monolayer, Δ*z*_layer_, Values Obtained for the Monolayer@Alumina Pairs Considered

layer	*E*_int_ (meV)	gap (eV)	*d*_layer‑supp_ (Å)	Δ*z*_layer_ (Å)
pG	600.72	0.0473	2.8942	0.0276
pBN	525.70	4.2868	2.4162	0.2979
ac1	846.99	0.2139	2.7168	0.0478
ac2	628.80	1.1720	2.7527	0.0877
alt1	738.28	2.0773	2.4495	0.2932
zz1	647.47	1.9110	2.6528	0.1294
i1	1100.23	1.4506	2.5532	0.5071
ac4	2579.67	0.2539	2.3635	0.2201
i2	2873.28	1.8144	2.1364	0.6605

**Figure 5:**
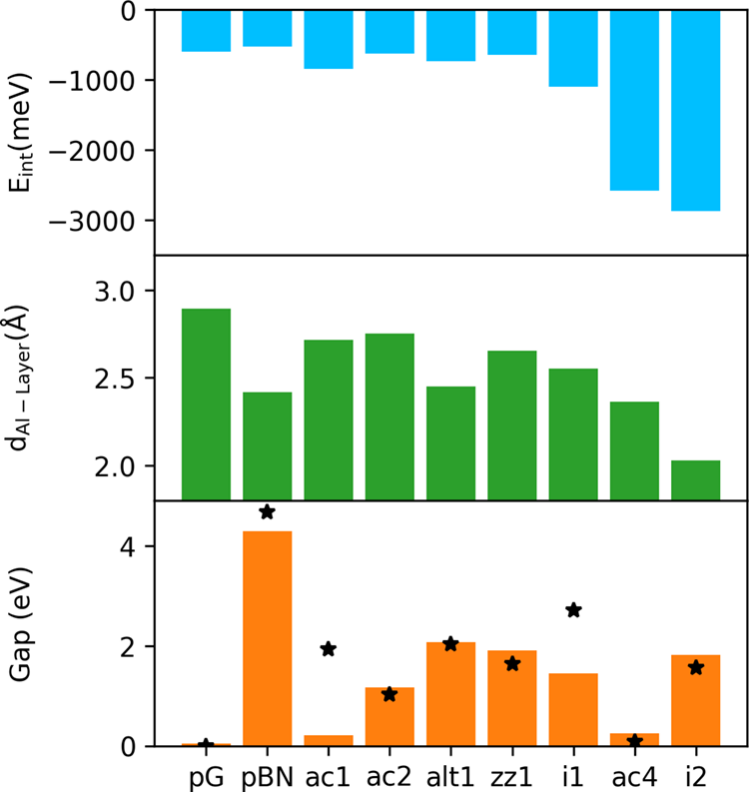
Interaction energy, distance, and gap for Monolayers@Alumina. Black stars show the band-gap value of the free monolayers.

**Figure 6:**
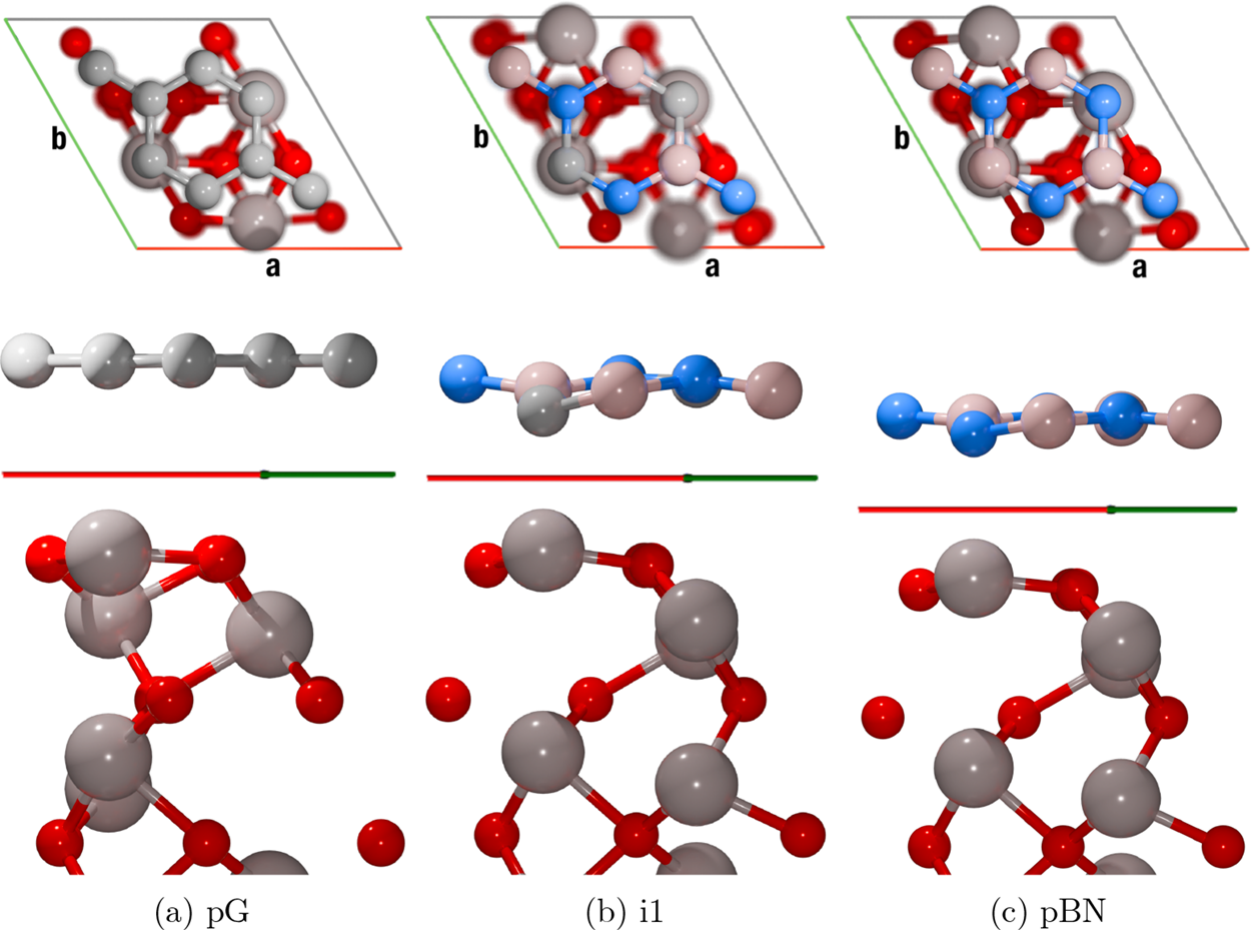
Top and lateral views of pG (a), i1 (b), and pBN (c) monolayers supported on (0001) α-Al_2_O_3_. In the lateral view, the *a*-axis (red) is parallel to the page plane, and the *b*-axis forms an angle of 60° with respect to it (consider a rotation of 180° of the top view along the *z*-axis). Atom color code: B, pink; C, gray; N, blue; O, red; and Al, light brown.

The EP maps for each Monolayer@Alumina were calculated (Figure 7), showing important differences with respect to the freestanding cases. These differences indicate a change in how the supported monolayers will interact with H_2_O molecules with respect to the freestanding ones.

**Figure 7:**
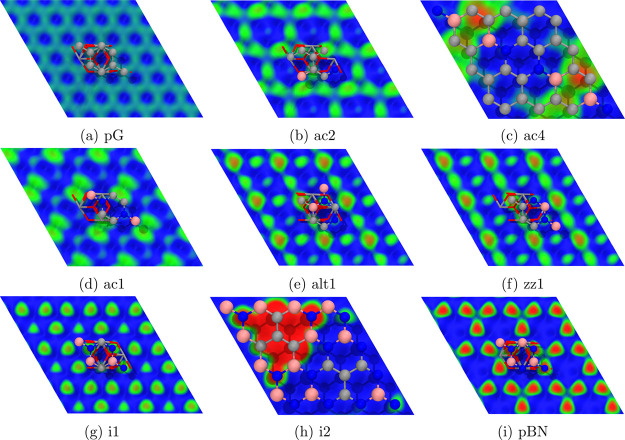
EP maps on top of the pristine (a, i) and BCN (b–h) monolayers supported on alumina, ρ_cut_ = 0.003, on the [0.01, 0.01] EP range (red-green-blue color scale). A fragment of each system is shown on top of the surface for reference. In the large unit cell systems (ac4 and i2), alumina atoms are not displayed for the sake of clarity. Atom representation: spheres for the monolayer (B, pink; C, gray; and N, blue) and sticks for the slab (O, red; and Al, gray). A larger format is available in the SI.

In line with this last statement, we calculated the *E*_int_ for H_2_O on alumina-supported pG, pBN, and i1, starting in each case from a geometry similar to that with the largest *E*_int_ for each system but only optimizing the H_2_O molecule coordinates. We chose these representative systems (the pristine ones plus i1, the one showing the most energetically favorable adsorption) to highlight the differences with the freestanding case while dealing with the increased computational cost: unit cells used here, large enough to avoid interaction between periodic water molecules, include 190 atoms. The predicted results, as shown in Figure 3, confirm how the interaction with the support changes the adsorptive nature of the examined monolayers. The overall effect is negative, i.e., the interaction is weaker, for pG and i1 (i.e., reducing the interaction), while it is positive for pBN with a slightly stronger interaction. For pBN and i1, we observe that the change of the *E*_int_ is consistent with the increment and reduction of the positive EP regions of the two supported monolayers.

While for both pBN and i1, the H_2_O-monolayer distances are close to the freestanding values, a considerable decrease is observed for pG. In the last section of the SI, we provide an analysis in terms of the electron density, ρ­(*r*), of both freestanding and supported pG. A deformation of ρ­(*r*) for the supported monolayer is clearly visible. This likely leads to a closer interaction with H_2_O but can also be ascribed to a less stable graphene layer that results in a weaker interaction. Such destabilization can be related to the lattice mismatch between the alumina slab and pG (1.73%), which imposes a contraction on the latter. This elastic constriction is also present in the other systems, as we report in the SI. Furthermore, apart from this spatial constraint, there might be some influence of the substrate, as charge redistribution effects can also be observed in the changes between freestanding and supported EP maps.

## Conclusions

Characterization of BCN systems at different compositions has been presented, showing characteristic spectral features that can be employed for experimental detection. The predicted EP distributions on the freestanding layers describe diverse interaction sites for the adsorption of interacting molecules. In the specific case of water, these interactions are predicted to be higher than the ones observed for the pristine systems.

The support of this BCN monolayer on top of (0001) α-Al_2_O_3_ leads to overall larger interaction energies, which increase with the amount of BN pairs in the system. The EP on top of the supported monolayer presents relevant changes with respect to the freestanding one, which translates to different interaction energies as we have shown for some systems.

While there is experimental evidence that graphene supported on top of (0001) α-Al_2_O_3_ can present a superstructure due to the cell mismatch between the support and the monolayer,^71^ and a similar situation might be expected for both pBN and BCN monolayers considered, some of the regions for such superstructures can probably be closely related to those discussed here. The present results thus provide evidence of how different BCN supported systems change their adsorption properties against the freestanding counterparts, and such information, along with the strength of the monolayer–support interaction, can be helpful for the development of coatings. As a perspective, this work sets the ground for the study of more complex chemical situations, such as the presence of defects in the alumina layer, higher adsorption coverage, and adsorption of different molecules than water.

## Supplementary Material



## Data Availability

Output files, which include the input file at their beginning, for the optimizations performed for the freestanding, supported, and H_2_O-adsorbed systems, and for the frequency calculations of freestanding monolayers, are publicly accessible at the NOMAD repository under 10.17172/NOMAD/2025.12.02-1. Basis sets used are available both at the repository and in the Supporting Information.

## Notes

Large-scale images for (i) freestanding unit cell systems, (ii) their electrostatic potential maps, (iii) water adsorption geometries for freestanding systems, (iv) electrostatic potential maps for alumina-supported systems, (v) layers supported on alumina, and (vi) water adsorption geometries for alumina-supported systems, along with additional details for the calculations, including basis sets, numerical parameters, surface formation energy data and methodology, and lattice mismatch values (PDF)
