# Supplementary material for: Water Adsorption Properties of Boron Carbonitride Monolayers: Effects of Substitution Patterns and Alumina Support
Source: ACS Omega. 2026 Feb 4;11(6):10172–80. doi: 10.1021/acsomega.5c11128 (PMC12917843; doi:10.1021/acsomega.5c11128)
Supplement: Supplementary file 1 [file ao5c11128_si_001.pdf]

Supplementary Material for: [Water Adsorption Properties of Boron Carbonitride Monolayers: Effects of Substitution Patterns and Alumina Support]

Marcos Rivera-Almazo, Bartolomeo Civalleri, and Lorenzo Maschio*

Dipartimento di Chimica, Università di Torino, Via P. Giuria 5, 10125 Torino, Italy

E-mail: lorenzo.maschio@unito.it

Freestanding monolayers unit cells

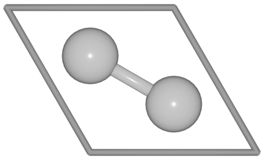

(a) Graphene

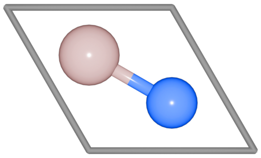

(b) h-BN

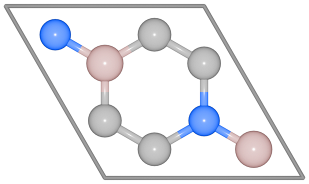

(c) ac1

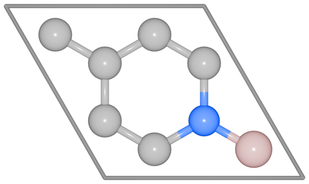

(d) ac2

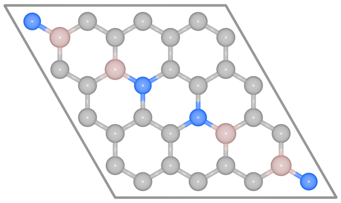

(e) ac4

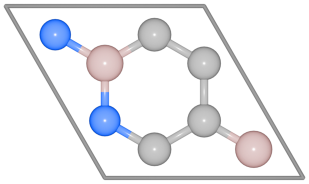

(f) alt1

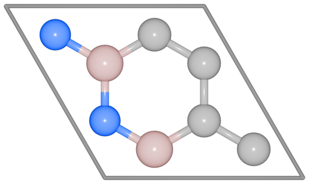

(g) zz1

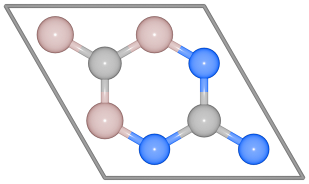

(h) i1

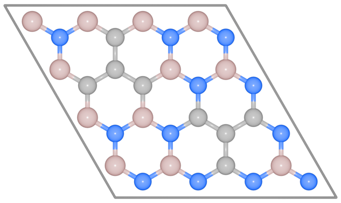

(i) i2

Figure S1: Unit cells of the freestanding pristine (a,b) and BCN systems (c-i).

Electrostatic potential maps for the freestanding layers

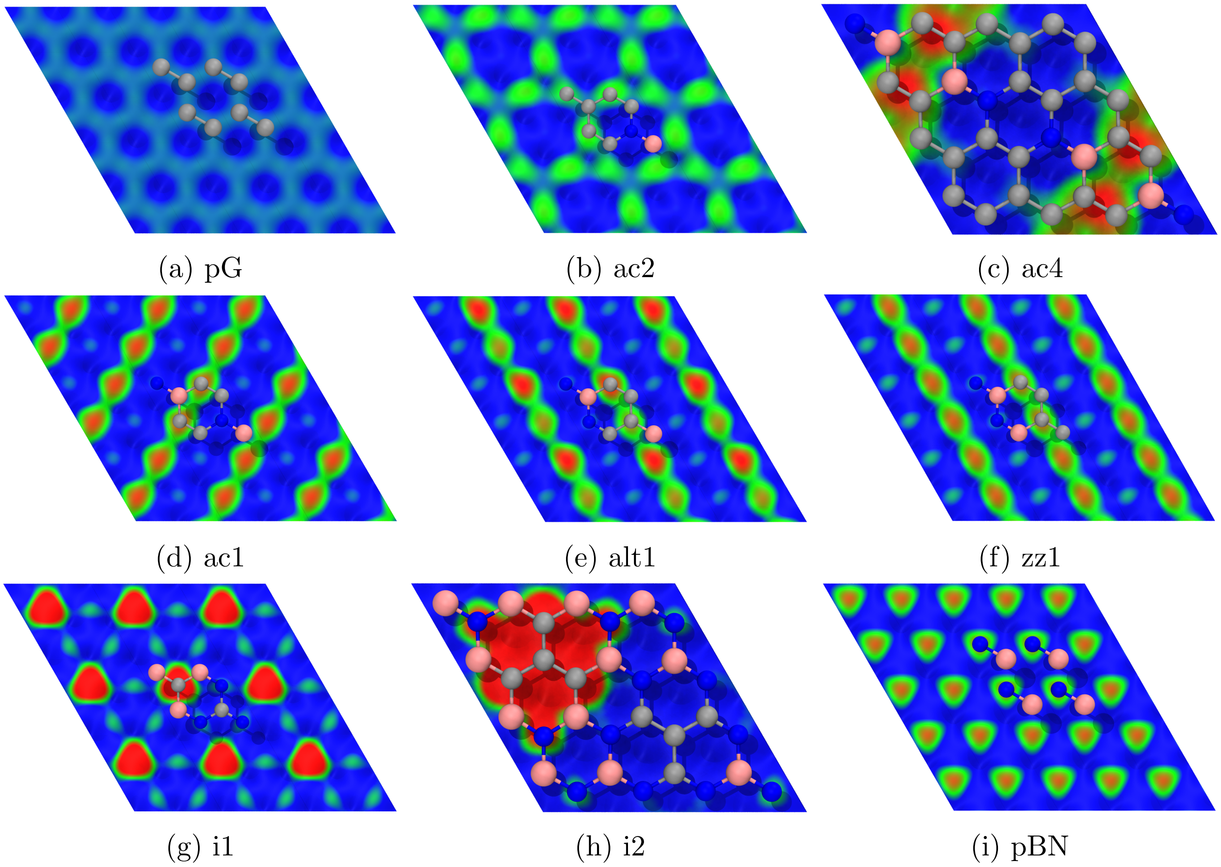

Figure S2: Electrostatic Potential (EP) maps on top of the freestanding pristine (a,i) and BCN (b-h) monolayers, $\rho_{cut} = 0.003$, on the [-0.01, 0.01] EP range (red-green-blue color scale). A fragment of each system is shown on top of the ρ surface as reference.

Adsorption geometries for H_2O@BCN

In this section the final calculated geometries for H_2O on the freestanding monolayers are shown, in each case following a decreasing order of E_{ads}.

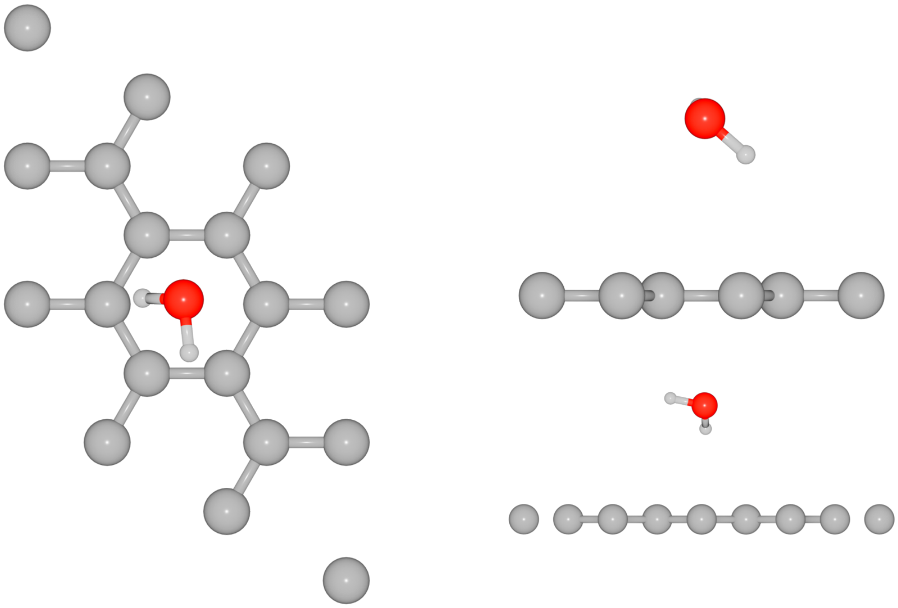

(a) C1: E_{ads}=-190.52 meV

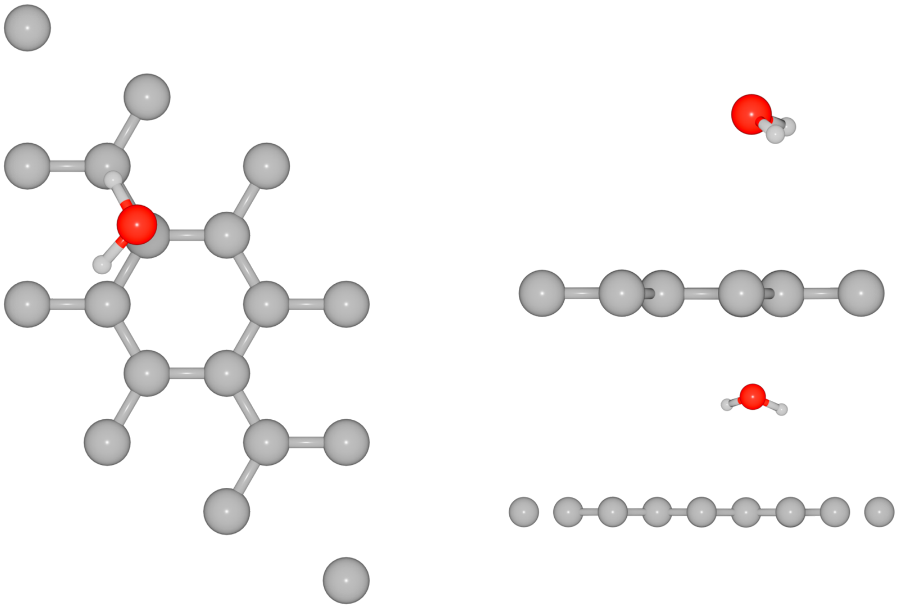

(b) C2: E_{ads}=-167.70 meV

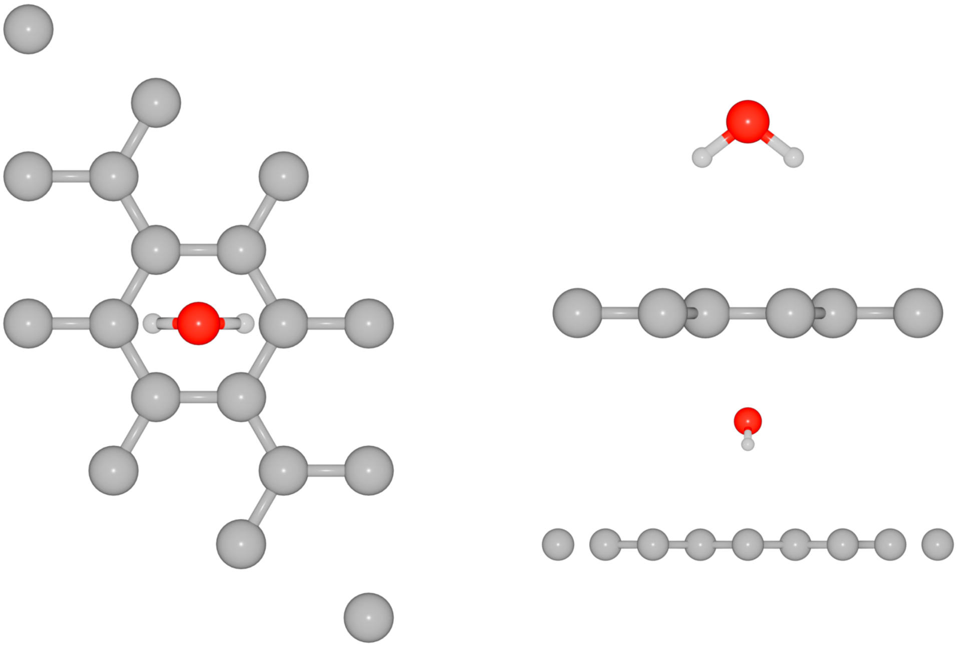

(c) C3: E_{ads}=-162.75 meV

Figure S3: Adsorption geometries C1 (a), C2 (b) and C3 (c), obtained for H_2O@pristine graphene.

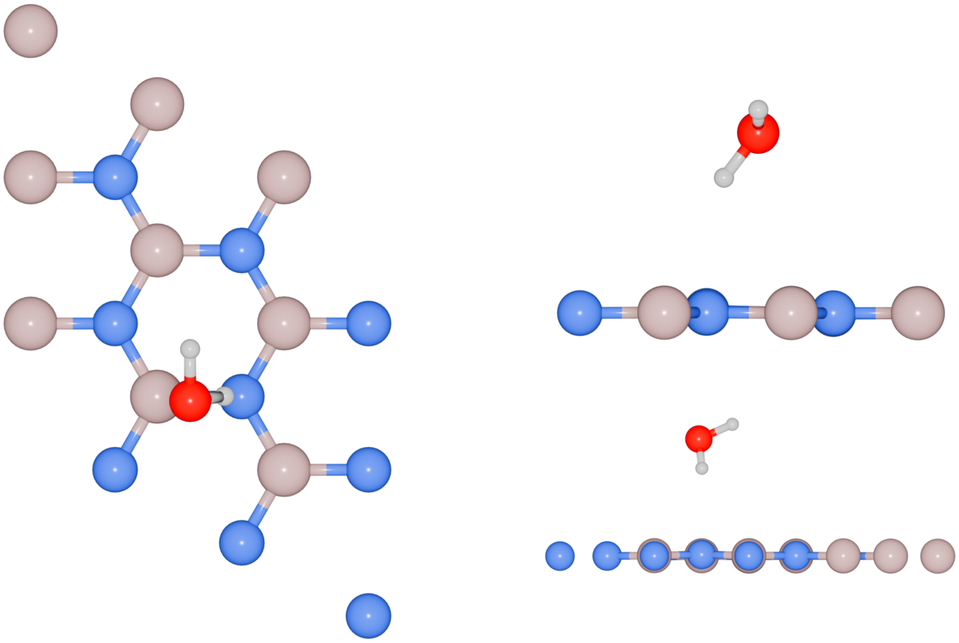

(a) C1: E_{ads}=-199.71 meV

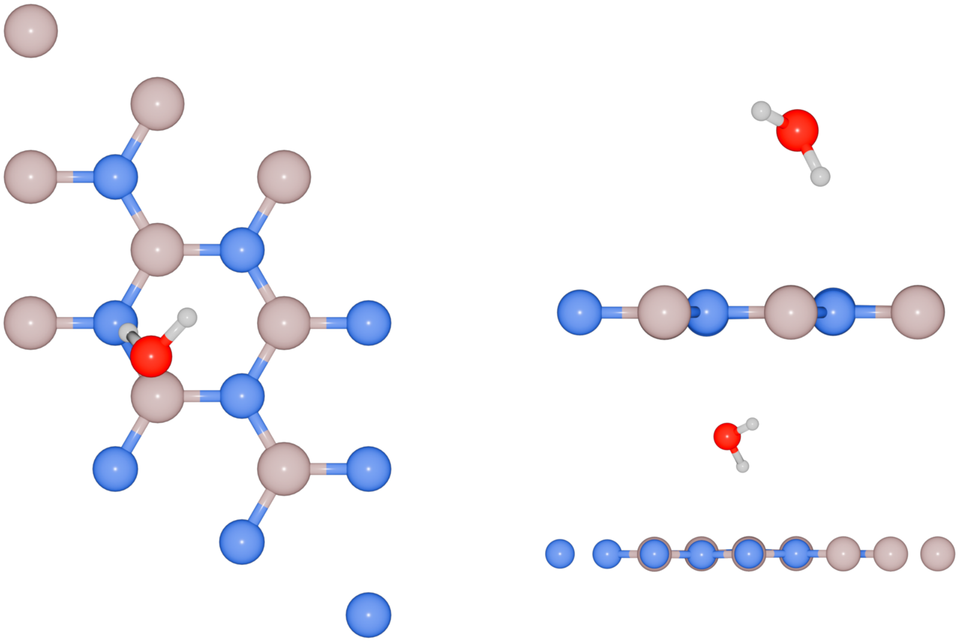

(b) C2: E_{ads}=-196.32 meV

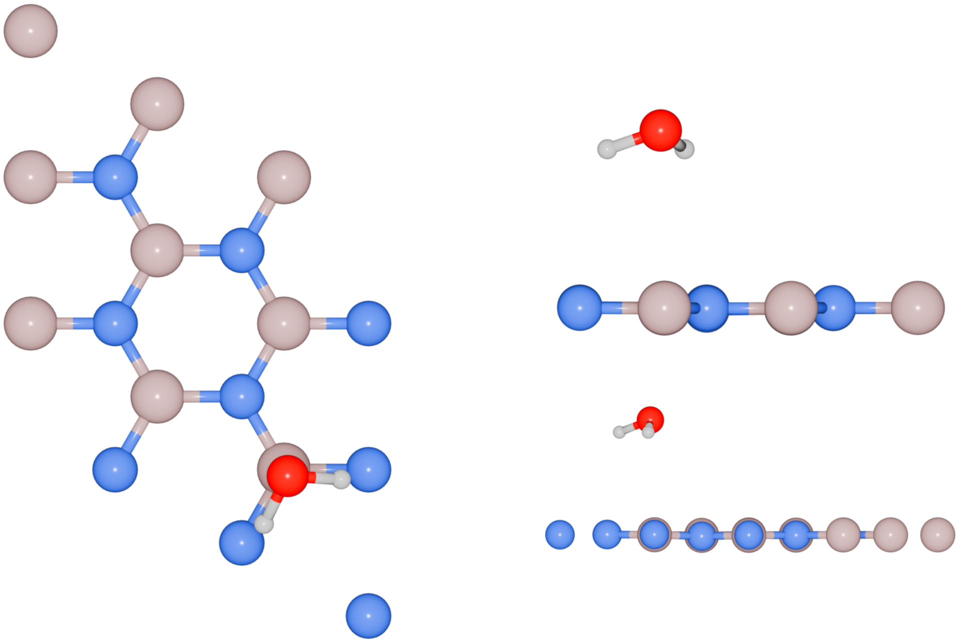

(c) C3: E_{ads}=-193.05 meV

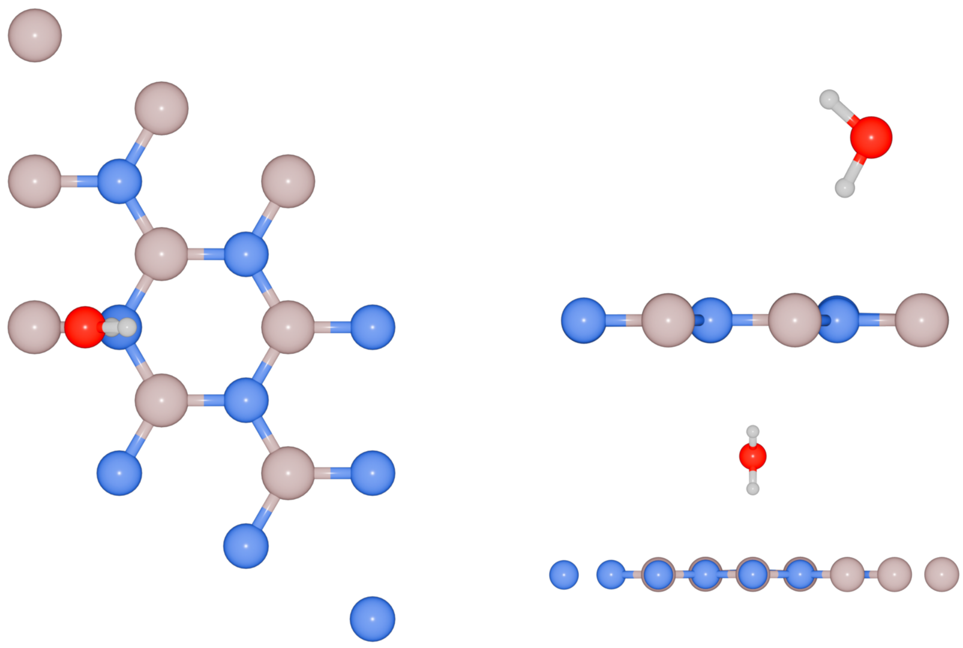

(d) C4: E_{ads}=-192.76 meV

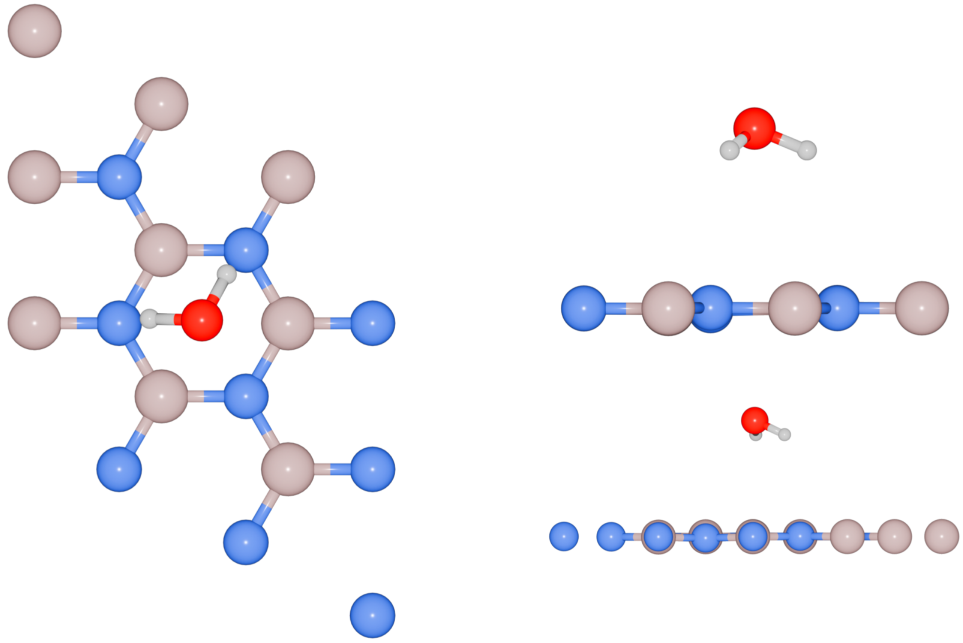

(e) C5: E_{ads}=-188.55 meV

Figure S4: Adsorption geometries C1 (a), C2 (b), C3 (c), C4 (d) and C5 (e), obtained for H_2O@pristine h-BN.

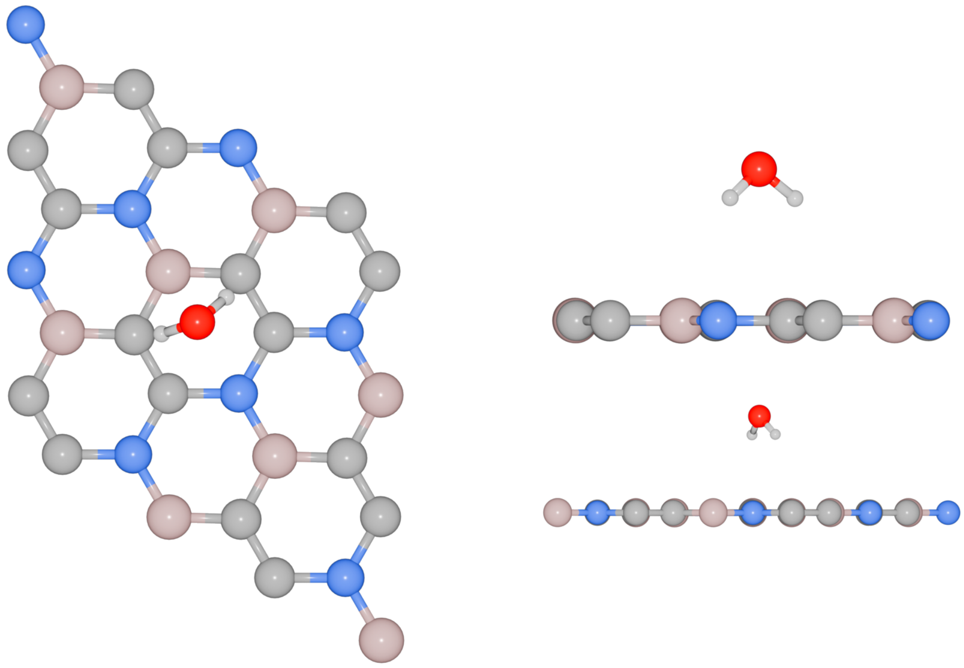

(a) C1: E_{ads}=-276.43 meV

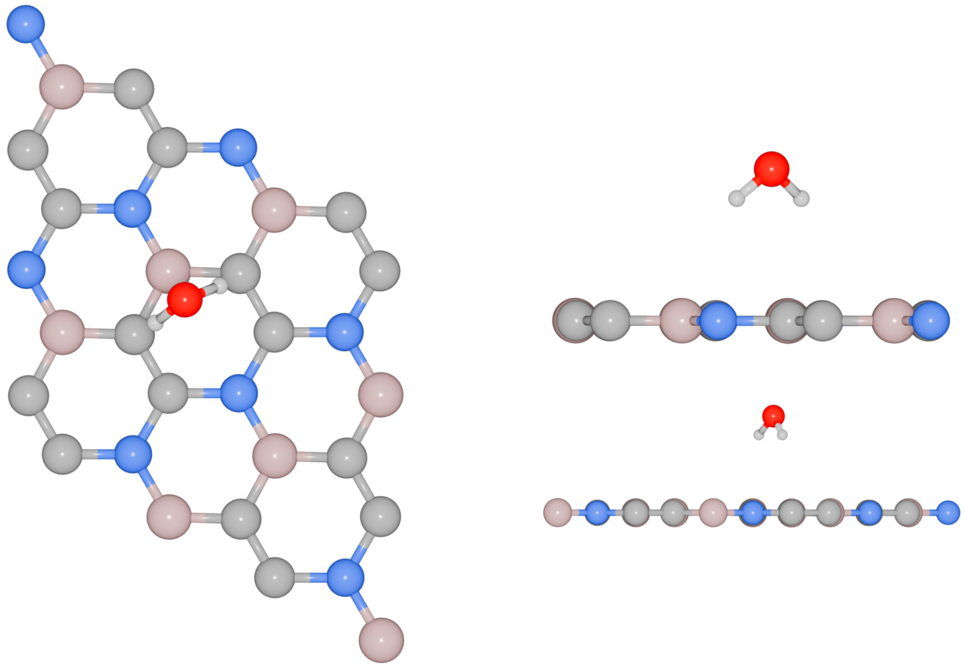

(b) C2: E_{ads}=-273.93 meV

Figure S5: Adsorption geometries C1 (a) and C2 (b), obtained for H_2O@ac1.

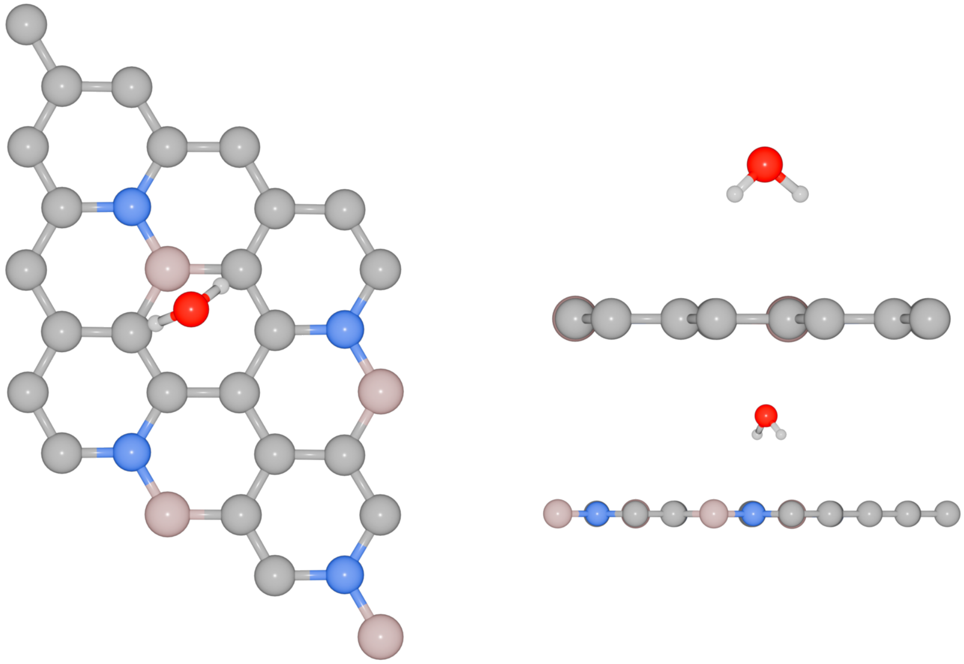

(a) C1: E_{ads}=-245.65 meV

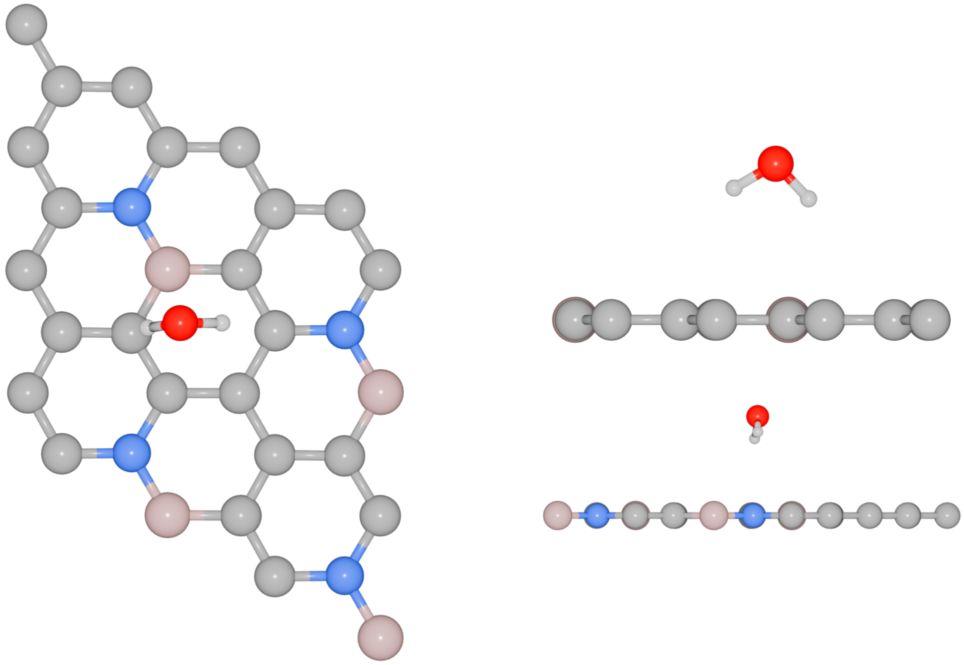

(b) C2: E_{ads}=-244.89 meV

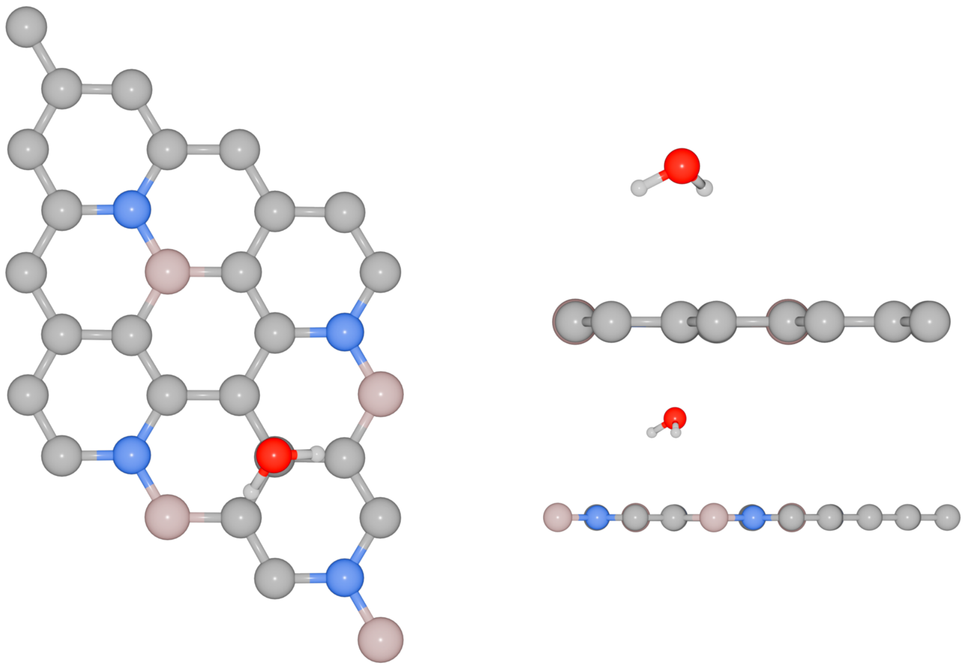

(c) C3: E_{ads}=-244.70 meV

Figure S6: Adsorption geometries C1 (a), C2 (b) and C3 (c), obtained for H_2O@ac2.

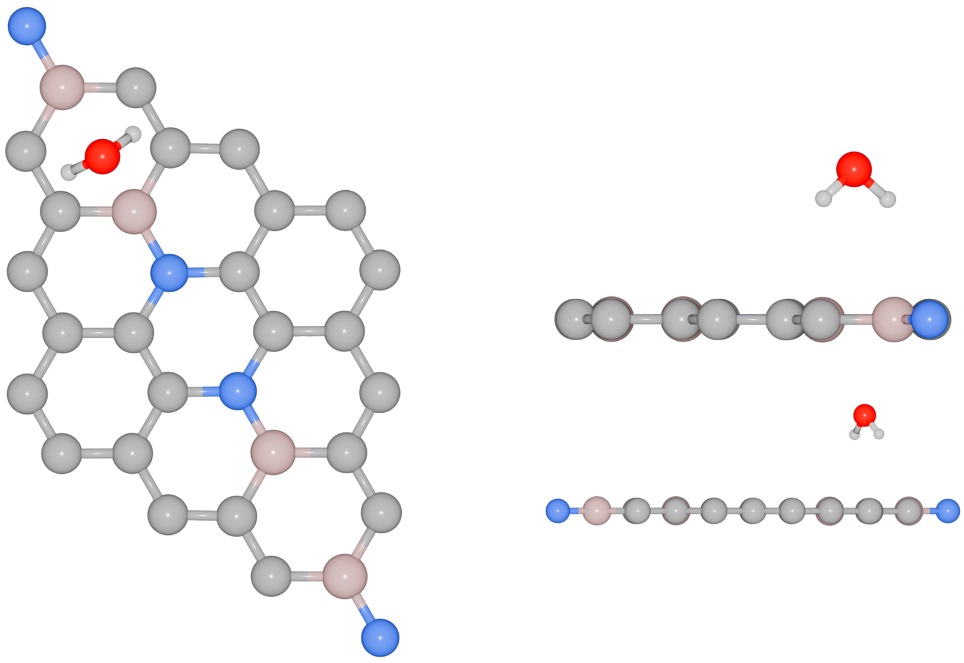

(a) C1: E_{ads}=-298.19 meV

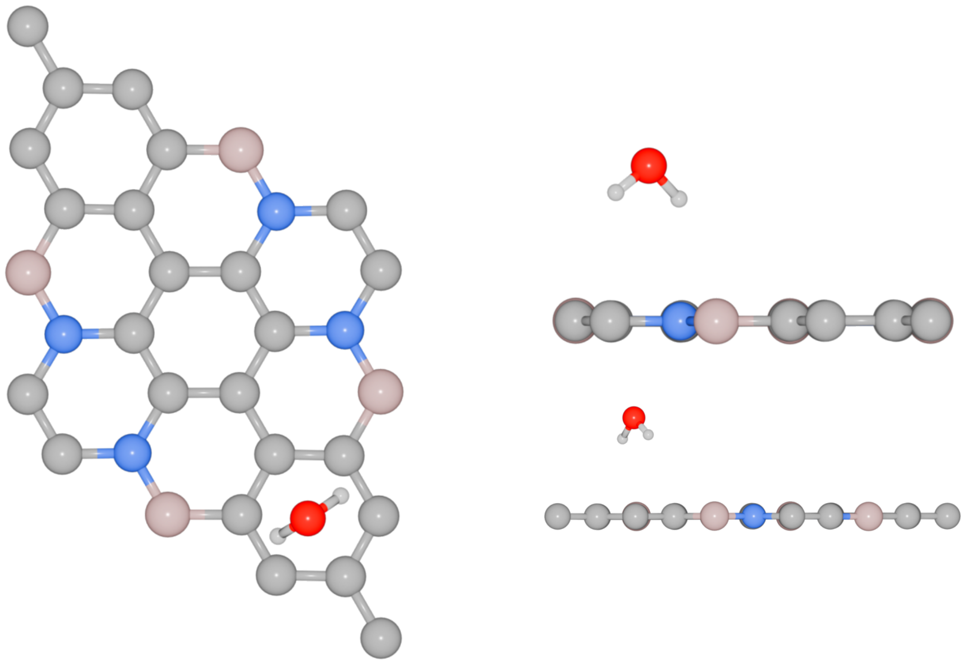

(b) C2: E_{ads}=-267.46 meV

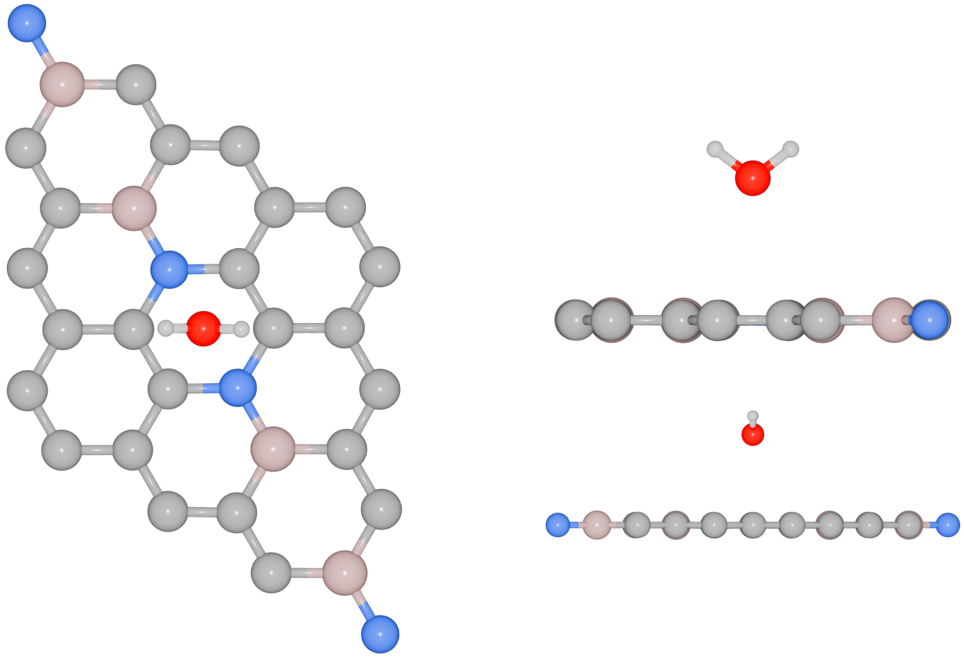

(c) C3: E_{ads}=-158.88 meV

Figure S7: Adsorption geometries C1 (a), C2 (b) and C3 (c), obtained for H_2O@ac4.

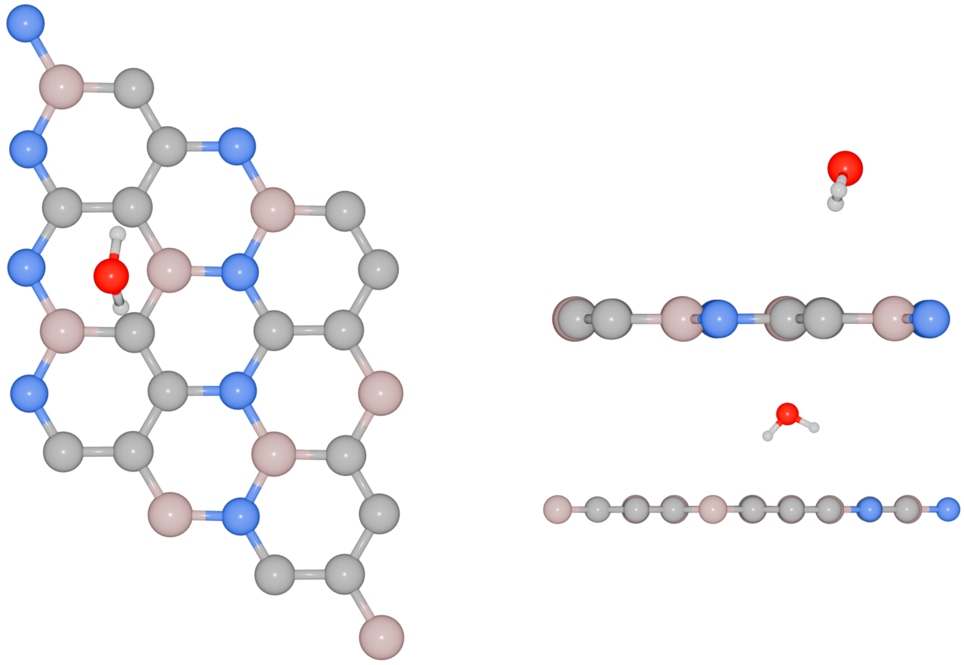

(a) C1: E_{ads}=-279.23 meV

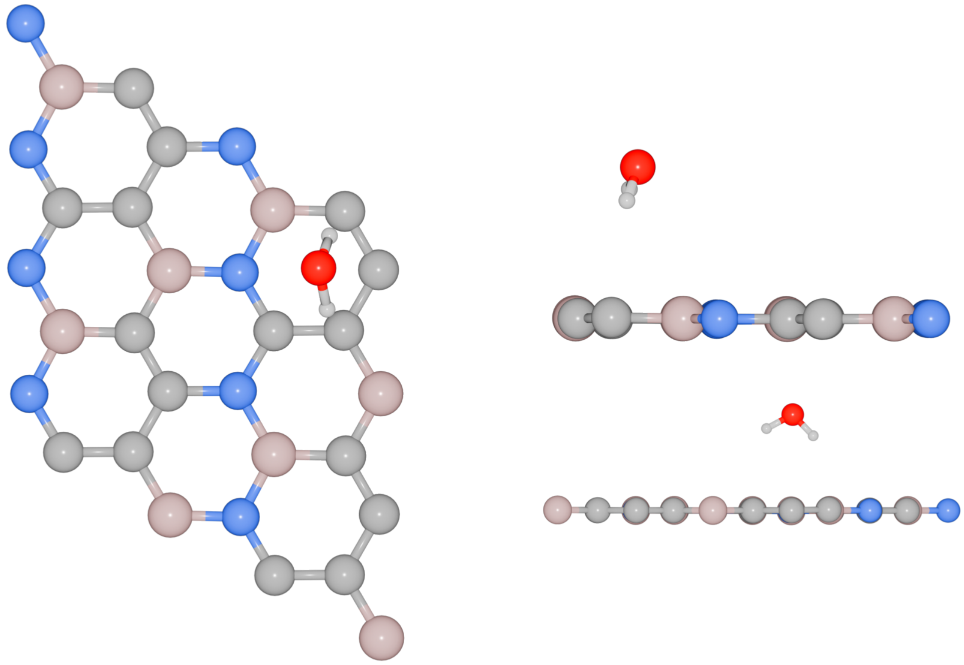

(b) C2: E_{ads}=-273.63 meV

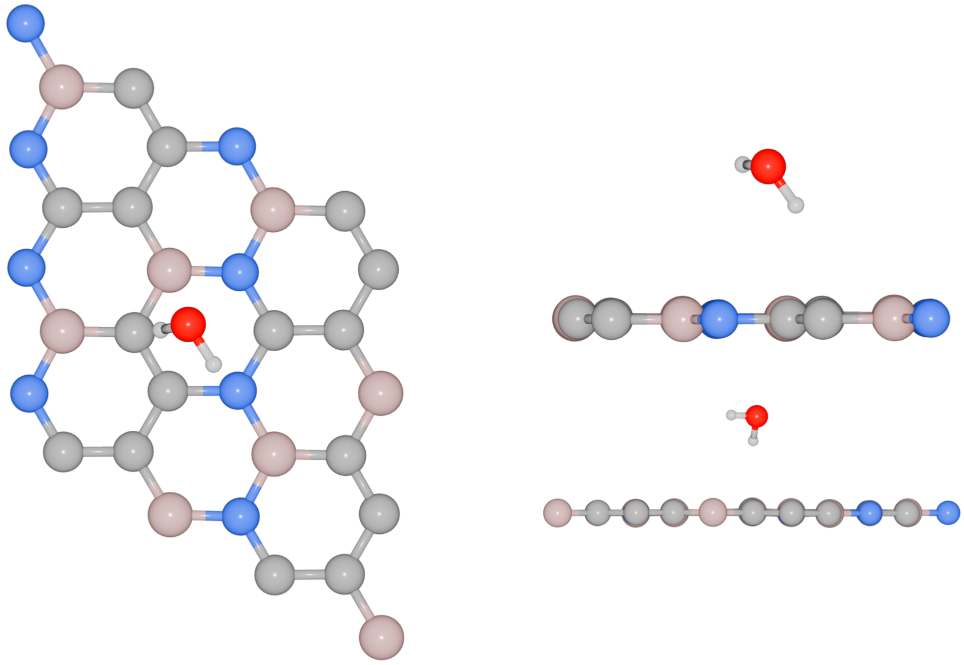

(c) C3: E_{ads}=-257.15 meV

Figure S8: Adsorption geometries C1 (a), C2 (b) and C3 (c), obtained for H_2O@alt1.

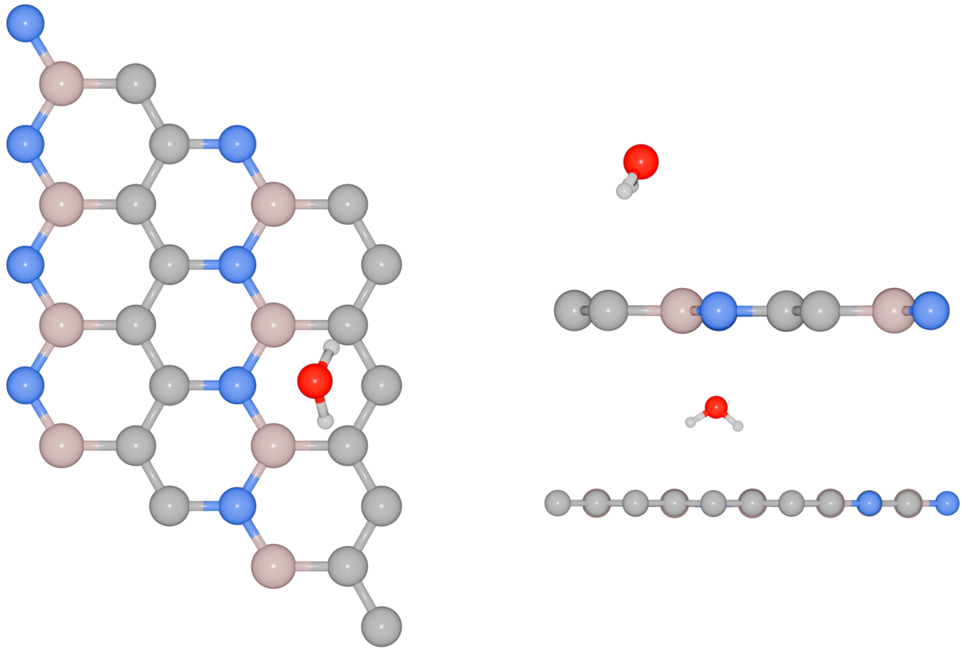

(a) C1: E_{ads}=-270.24 meV

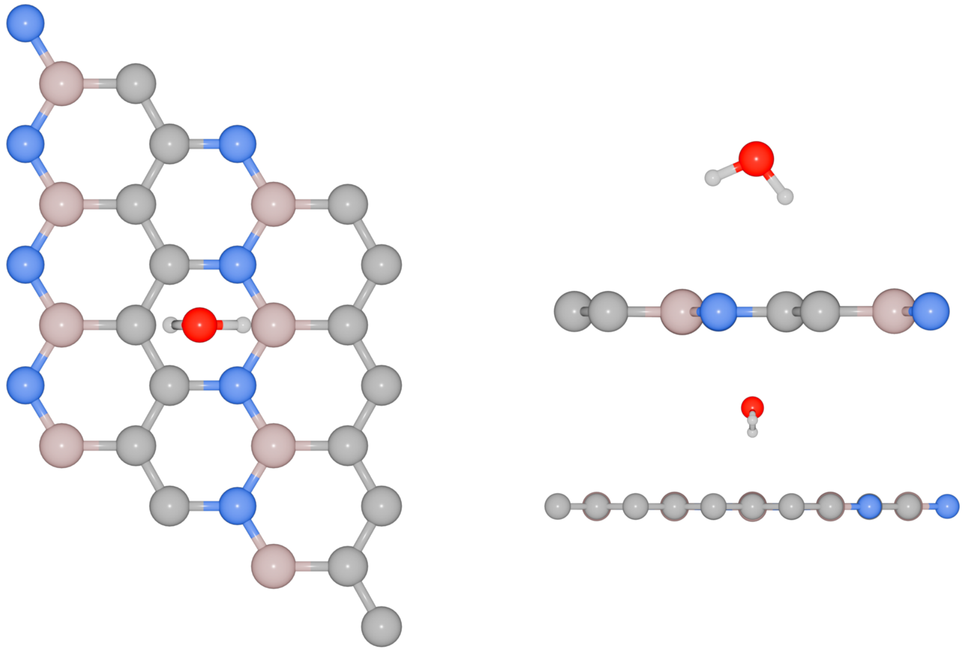

(b) C2: E_{ads}=-238.21 meV

Figure S9: Adsorption geometries C1 (a) and C2 (b), obtained for H_2O@zz1.

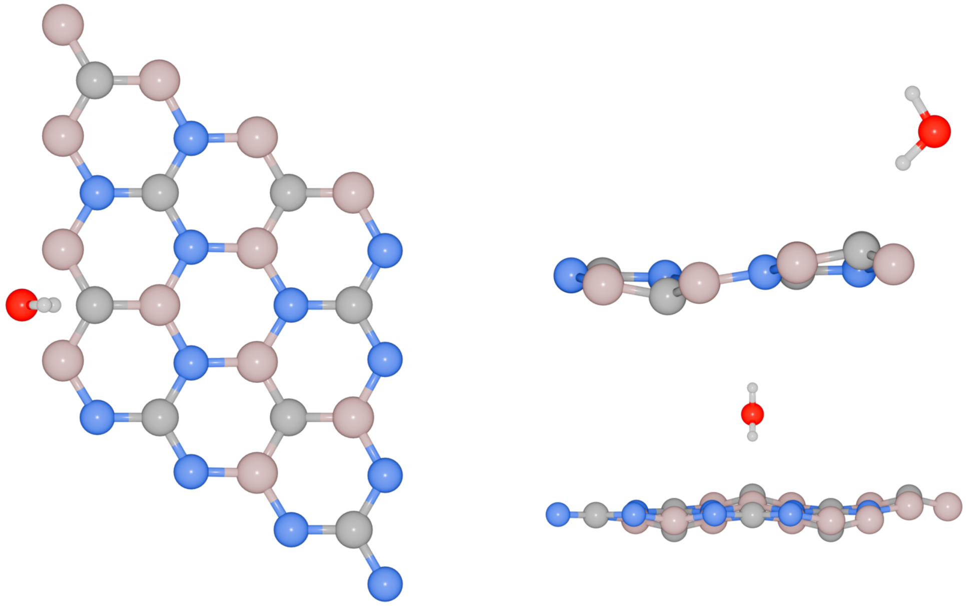

(a) C1: E_{ads}=-460.75 meV

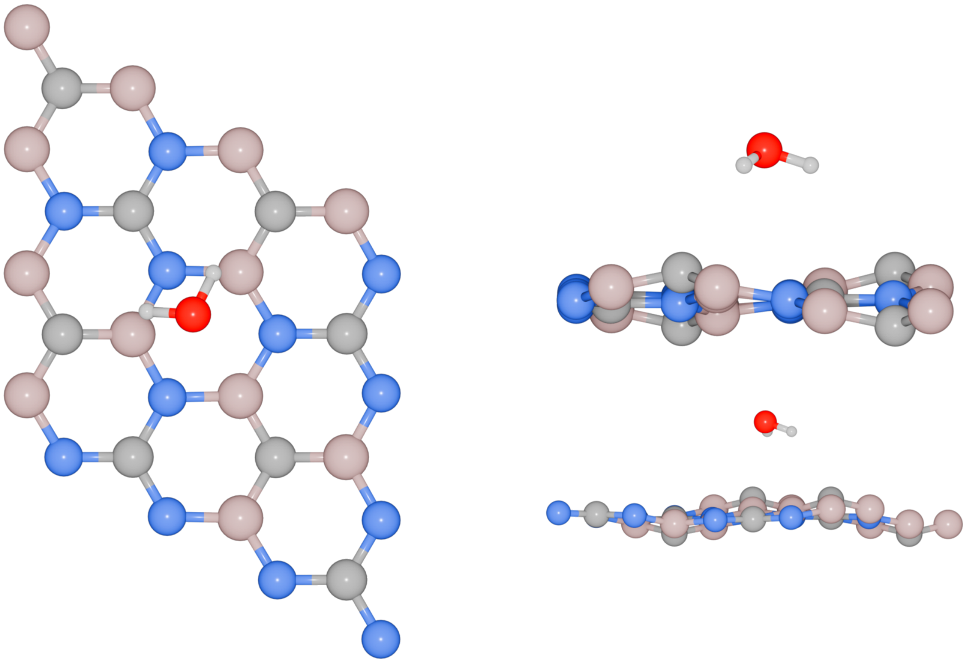

(a) C2: E_{ads}=-455.32 meV

Figure S11: Adsorption geometries C1 (a) and C2 (b), obtained for H_2O@i1.

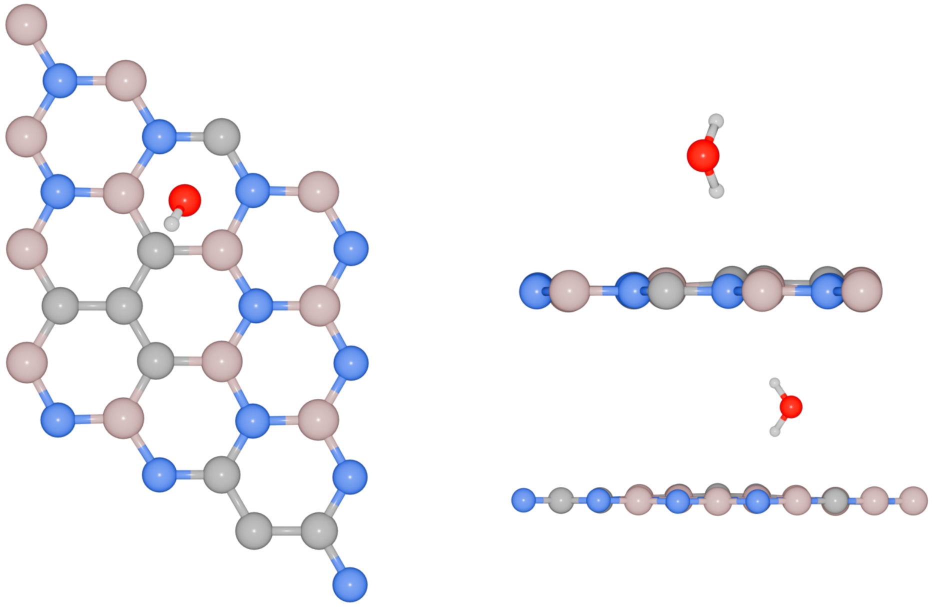

(a) C1: E_{ads}=-382.23 meV

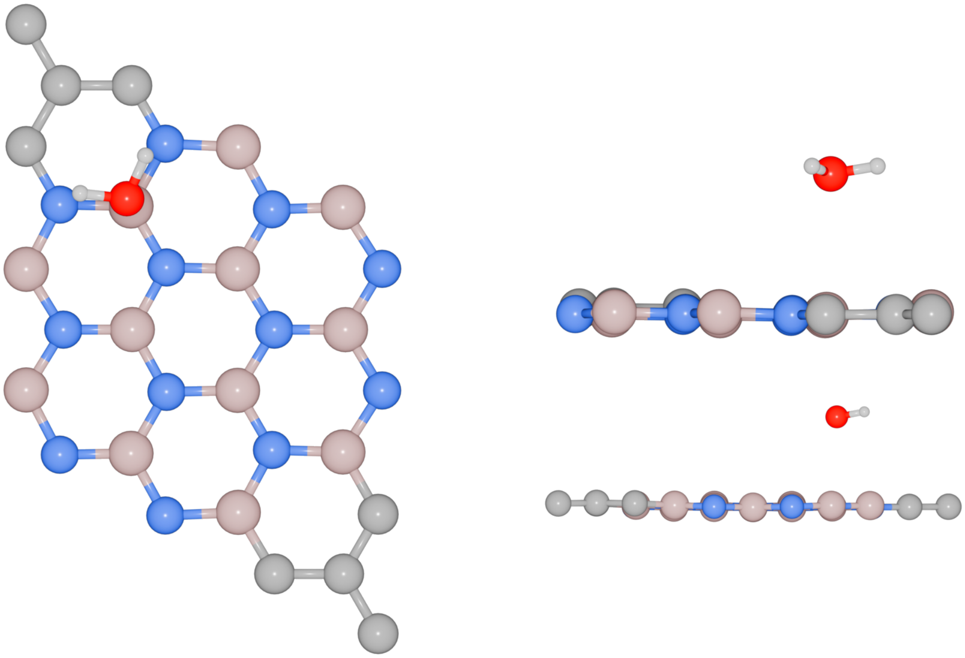

(b) C2: E_{ads}=-204.60 meV

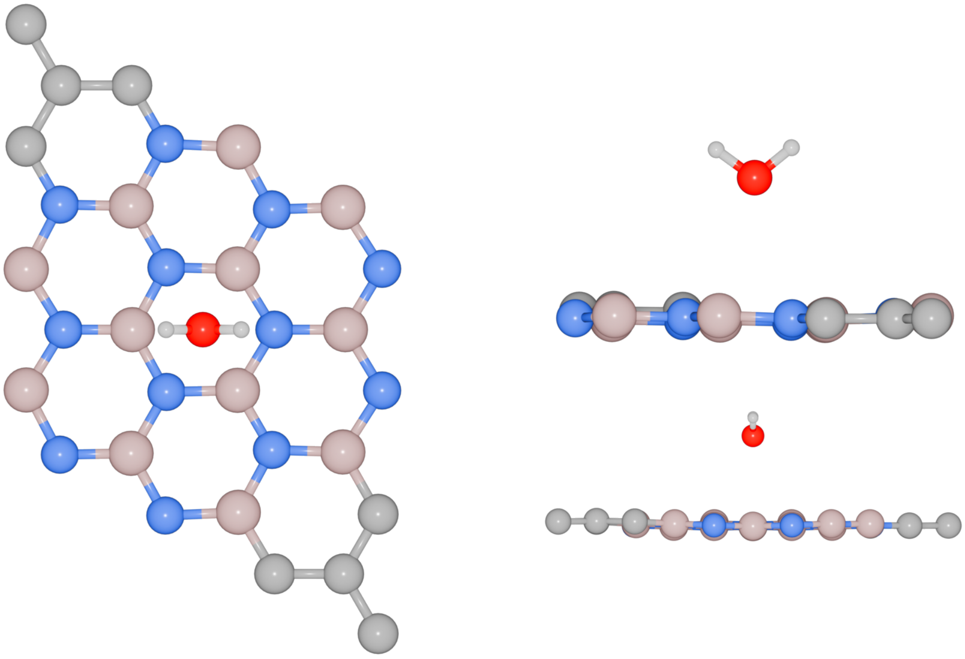

(c) C3: E_{ads}=-137.59 meV

Figure S12: Adsorption geometries C1 (a), C2 (b) and C3 (c), obtained for H_2O@i2.

Electrostatic potential maps for the Alumina-supported layers

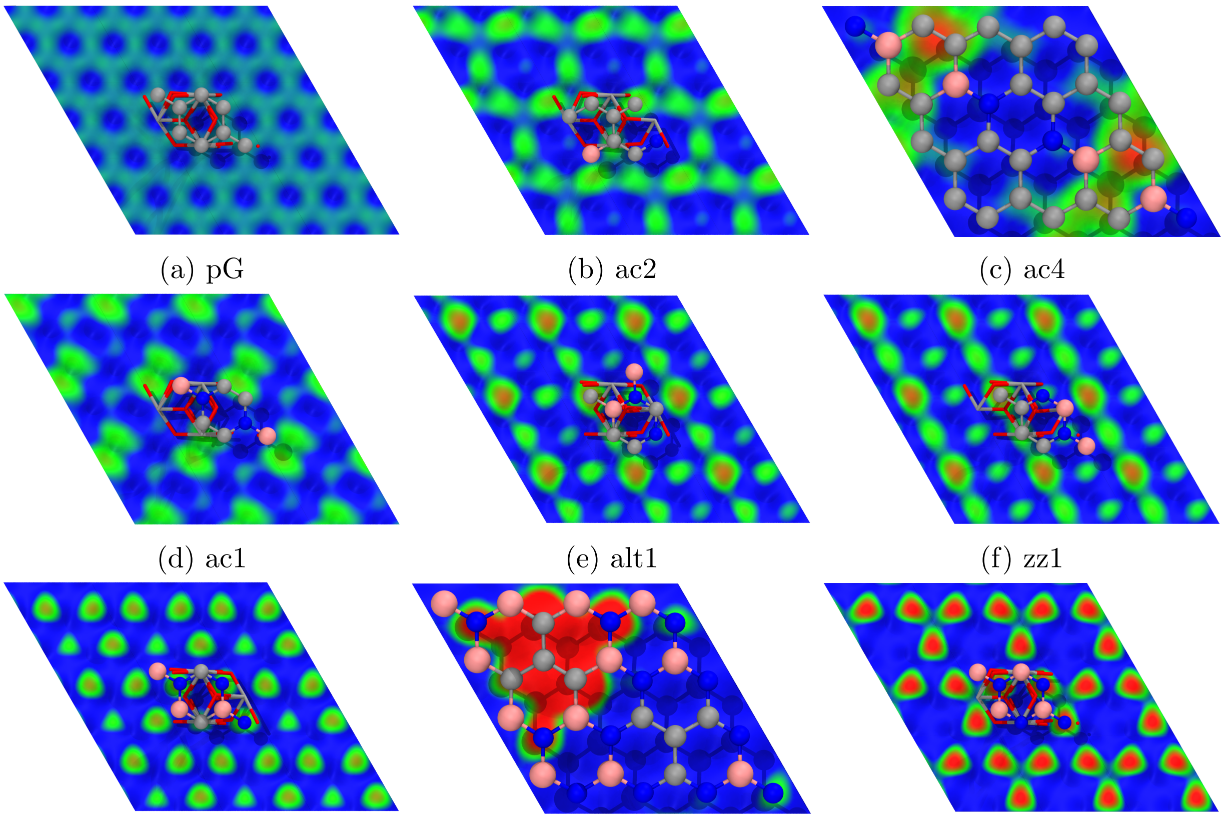

(a) pG (b) ac2 (c) ac4
(d) ac1 (e) alt1 (f) zz1
(g) i1 (h) i2 (i) pBN

Figure S13: EP maps on top of the pristine (a,i) and BCN (b-h) monolayers supported on alumina, $\rho_{cut} = 0.003$, on the [-0.01, 0.01] EP range (red-green-blue color scale). A fragment of each system is shown on top of the surface for reference. In the large unit cell systems (ac4 and i2), alumina atoms are not displayed for clarity.

Adsorption geometries for BCN@Alumina

In this section the final optimized geometries for the freestanding monolayers on top of the (0001) α-Al_2O_3 surface model are shown.

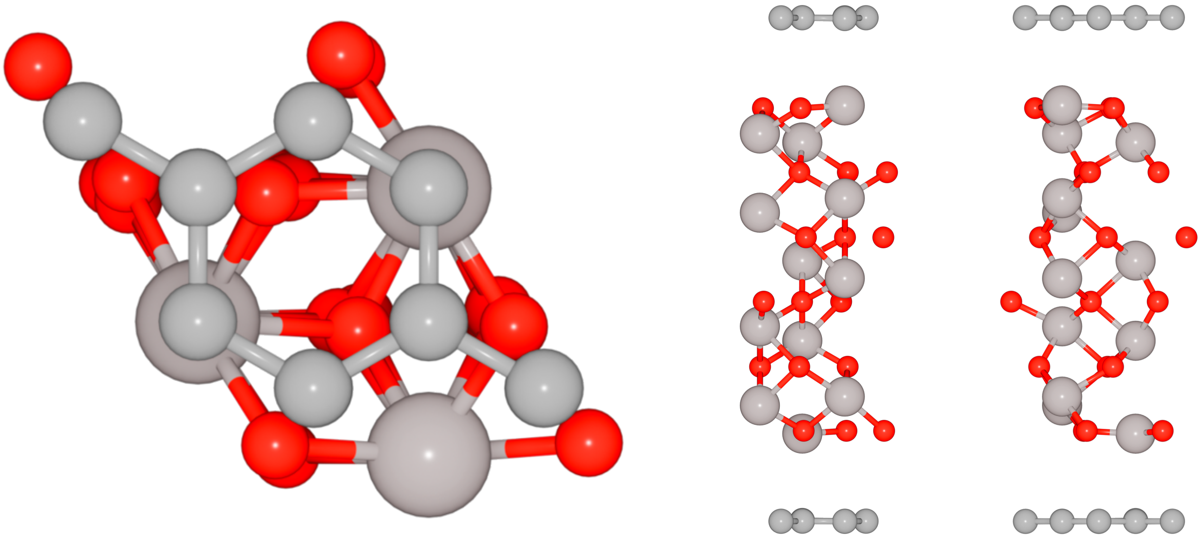

Figure S14: pristine graphene

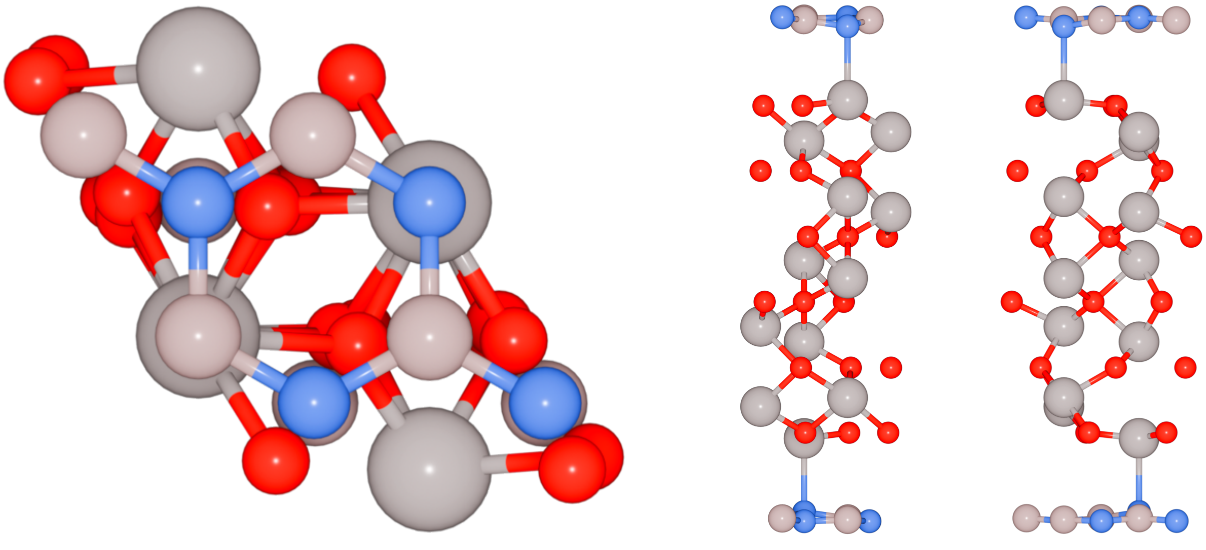

Figure S15: pristine h-BN

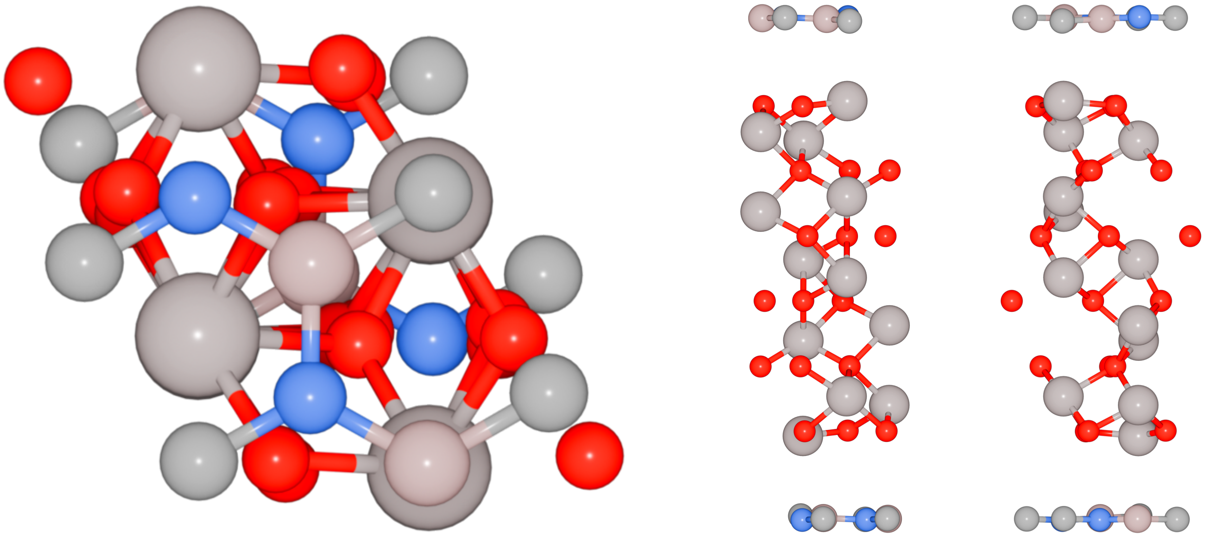

Figure S16: ac1

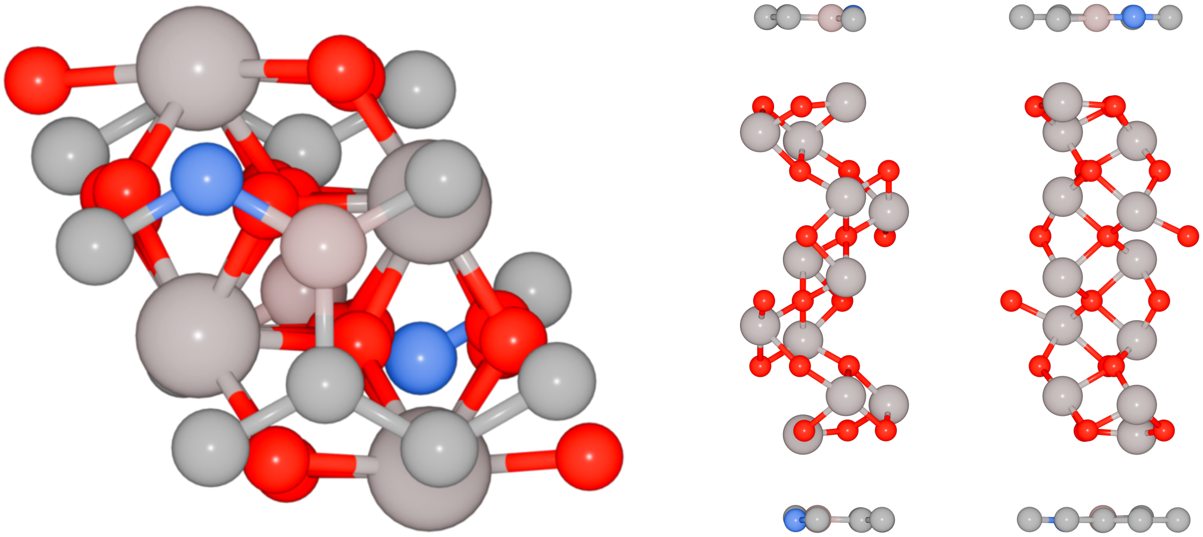

Figure S17: ac2

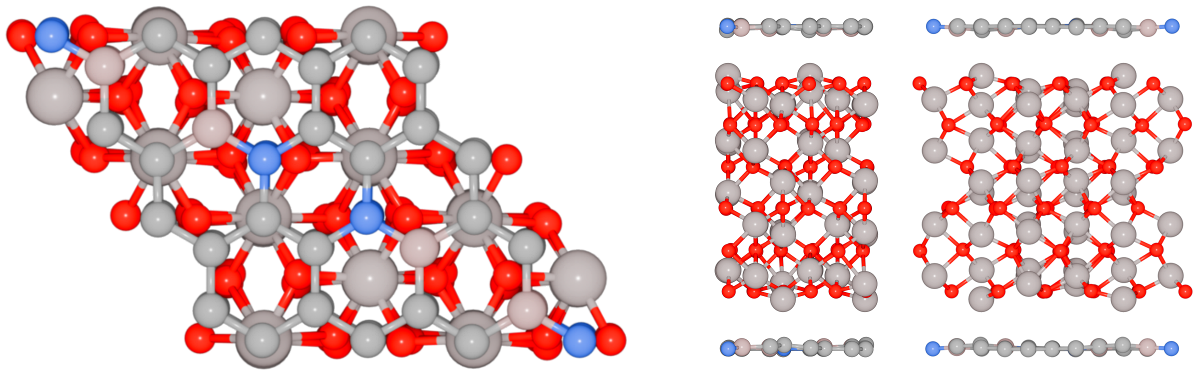

Figure S18: ac4

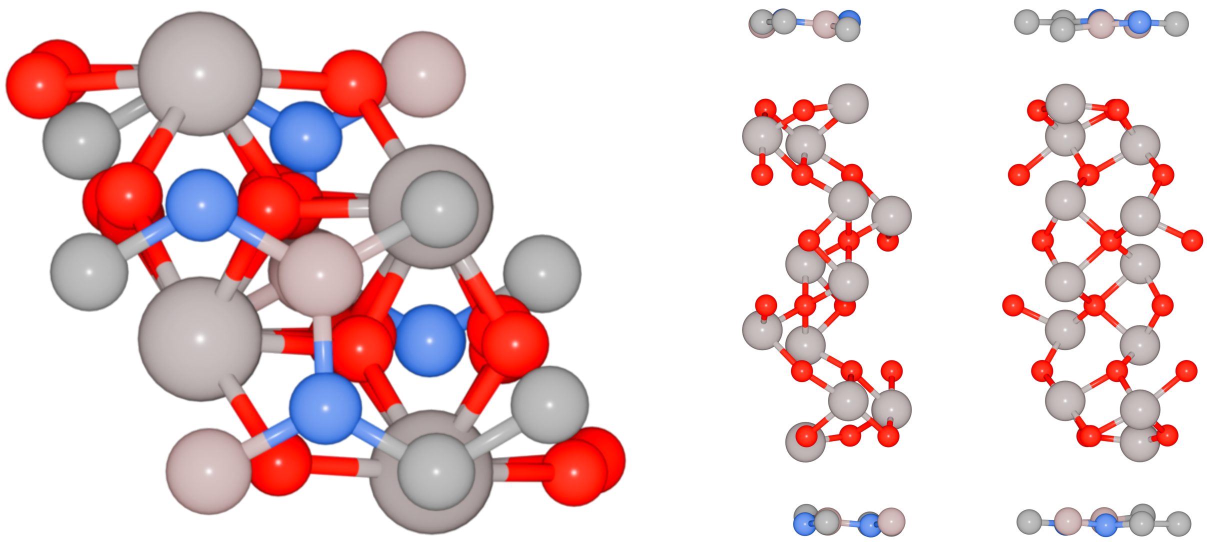

Figure S19: alt1

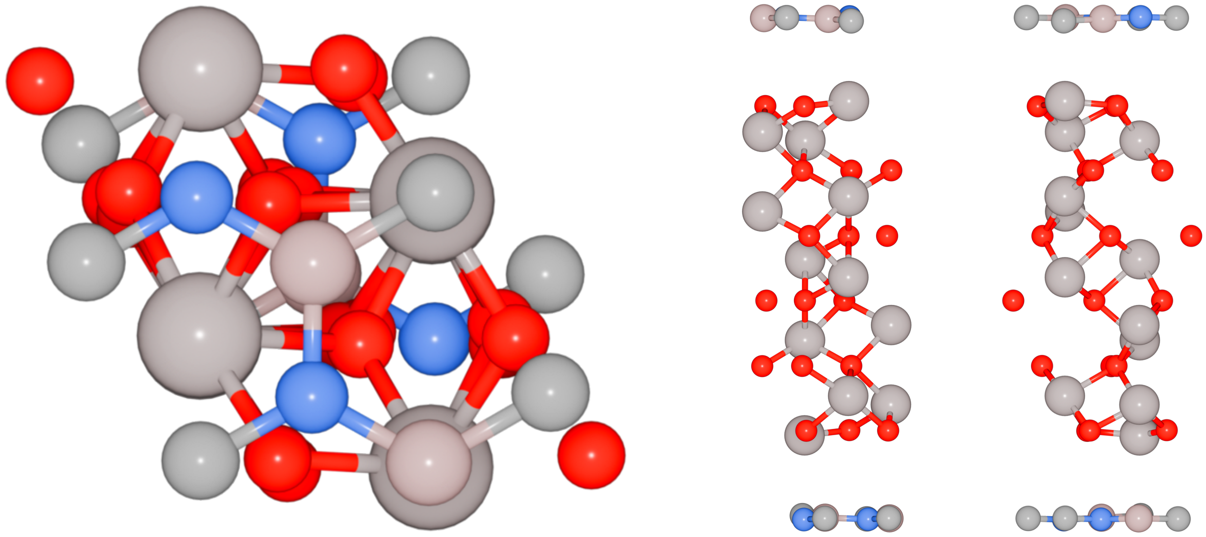

Figure S20: zz1

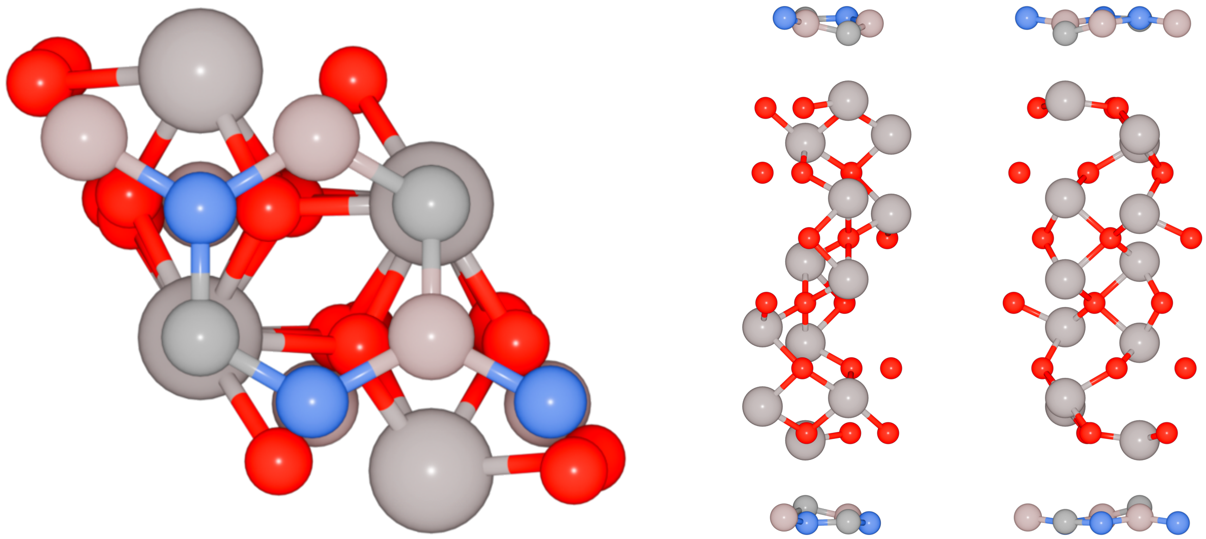

Figure S21: i1

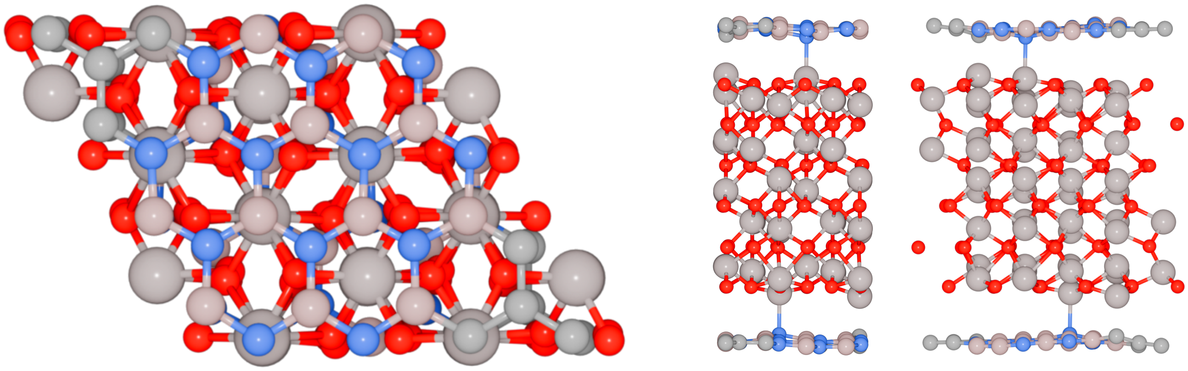

Figure S22: i2

Adsorption geometries for H_2O@Alumina-supported BCN

In this section the optimized geometries for H_2O on top of Alumina-supported graphene, h-BN and the BCN i1 pattern are shown.

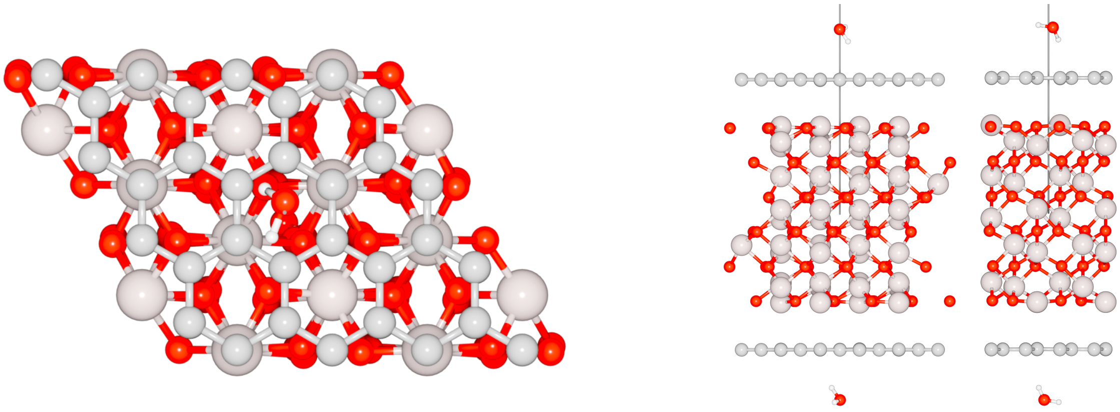

Figure S23: pristine graphene

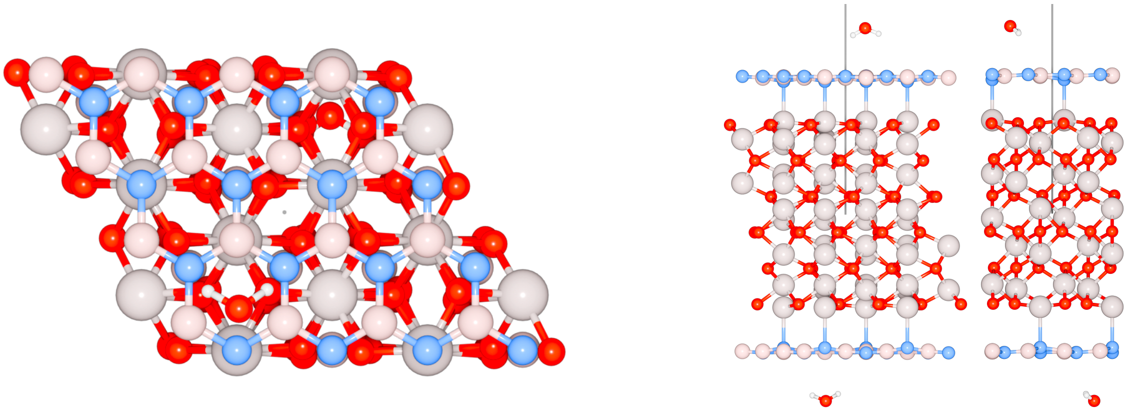

Figure S24: pristine h-BN

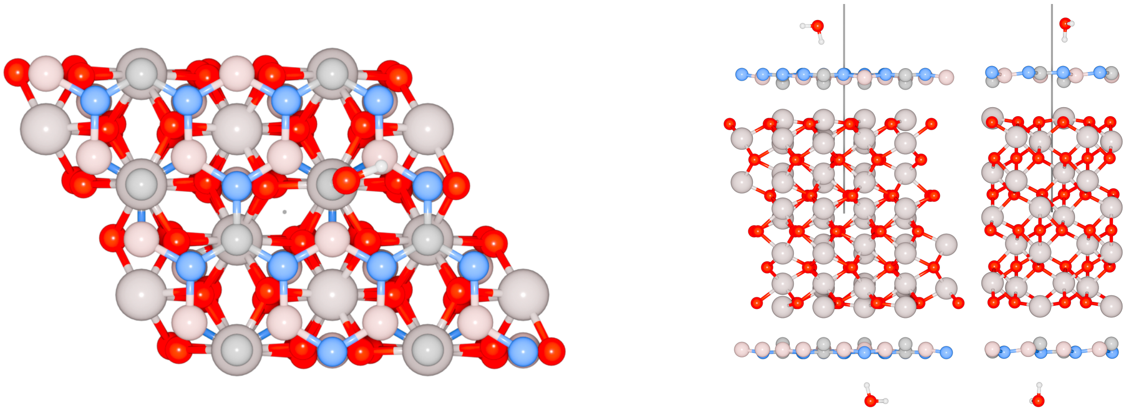

Figure S25: i1

CRYSTAL calculations settings

Basis sets

The content of the BASISSETS.DAT file, condensing the different basis sets employed, is shown below. Basis for B, C and N (5, 6 and 7 atomic numbers) were optimized for their most common corresponding elemental solid, using def2-TZVP as starting reference. For B and N, a subsequent optimization in the h-BN system was performed. All these basis set optimizations were done employing the BDIIS algorithm implemented in CRYSTAL23.[1] For Al and O atoms belonging to the (0001) α-$Al_2 0_3$ slab part POB-TZVP basis were selected.[2] For H and O of the H_2O molecule, the def2-TZVP molecular basis[3–5] was chosen (atomic labels 101 and 108 in the BASISSETS.DAT file).

```
#BASIS

Basis sets for BCN systems supported in alpha-Alumina

5 10

0 0  6  2. 1.

      8.56486606870E+03   2.28371981550E-04

      1.28415162630E+03   1.76825764470E-03
```

```
      2.92278716040E+02   9.14070805160E-03
      8.27754691760E+01   3.63426389890E-02
      2.70179392690E+01   1.10634584410E-01
      9.81496196600E+00   2.33673443210E-01
0 0  2  2. 1.
      3.93185590590E+00   4.18187779780E-01
      1.65955997120E+00   2.23254737980E-01
0 0  1  0. 1.
      8.66844775290E-01   1.00000000000E+00
0 0  1  0. 1.
      4.93020846128E-01   1.00000000000E+00
0 0  1  0. 1.
      1.81494022662E-01   1.00000000000E+00
0 2  4  1. 1.
      2.24538758030E+01   5.02655751790E-03
      5.10450583300E+00   3.28017389650E-02
      1.49860813440E+00   1.31512307680E-01
      5.09278313150E-01   3.31971677690E-01
0 2  1  0. 1.
      9.79162218583E-01   1.00000000000E+00
0 2  1  0. 1.
      1.59756066721E-01   1.00000000000E+00
0 3  1  0. 1.
      6.74578611498E-01   1.00000000000E+00
0 4  1  0. 1.
      4.96872101940E-01   1.00000000000E+00
7 11
```

```
0 0  6  2. 1.
      1.97308006470E+04   2.18879849910E-04
      2.95789587450E+03   1.69607088030E-03
      6.73221335950E+02   8.79546035380E-03
      1.90682494940E+02   3.53593826050E-02
      6.22954418980E+01   1.10957892170E-01
      2.26541611820E+01   2.49829725520E-01
0 0  2  2. 1.
      8.97914774280E+00   4.06238961480E-01
      3.68630023700E+00   2.43382171760E-01
0 0  1  0. 1.
      9.86073621989E-01   1.00000000000E+00
0 0  1  0. 1.
      4.33621361542E-01   1.00000000000E+00
0 0  1  0. 1.
      1.03005200572E-01   1.00000000000E+00
0 2  4  3. 1.
      4.92003805100E+01   5.55524167510E-03
      1.13467905370E+01   3.80523797230E-02
      3.42739724110E+00   1.49536710290E-01
      1.17855251340E+00   3.49493052300E-01
0 2  1  0. 1.
      4.06669162933E-01   1.00000000000E+00
0 2  1  0. 1.
      1.43226692228E-01   1.00000000000E+00
0 3  1  0. 1.
      1.72446376964E+00   1.00000000000E+00
```

```
0 3  1  0. 1.
      5.62174616901E-01   1.00000000000E+00
0 4  1  0. 1.
      1.38944141131E+00   1.00000000000E+00
  6 11
0 0  6  2. 1.
   1.35753496820E+04   2.22458143520E-04
   2.03523336800E+03   1.72327382520E-03
   4.63225623590E+02   8.92557153140E-03
   1.31200195980E+02   3.57279845020E-02
   4.28530158910E+01   1.10762599310E-01
   1.55841857660E+01   2.42956276260E-01
0 0  2  2. 1.
   6.20671385080E+00   4.14402634480E-01
   2.57648965270E+00   2.37449686550E-01
0 0  1  0. 1.
   9.96164942002E-01   1.00000000000E+00
0 0  1  0. 1.
   6.38373126590E-01   1.00000000000E+00
0 0  1  0. 1.
   2.31166652661E-01   1.00000000000E+00
0 2  4  2. 1.
   3.46972322440E+01   5.33336578050E-03
   7.95826228260E+00   3.58641090920E-02
   2.37808268830E+00   1.42158733290E-01
   8.14332081830E-01   3.42704718450E-01
0 2  1  0. 1.
```

```
   3.84832397891E-01   1.00000000000E+00
0 2  1  0. 1.
   1.87899705316E-01   1.00000000000E+00
0 3  1  0. 1.
   1.01061584864E+00   1.00000000000E+00
0 3  1  0. 1.
   6.14506968465E-01   1.00000000000E+00
0 4  1  0. 1.
   7.55573159273E-01   1.00000000000E+00
101 4
0 0 3 1 1.0
     34.0613410              0.60251978D-02
      5.1235746              0.45021094D-01
      1.1646626              0.20189726
0 0 1 0 1.0
      0.32723041             1.0000000
0 0 1 0 1.0
      0.10307241             1.0000000
0 2 1 0 1.0
      0.8000000              1.0000000
108 11
0 0 6 2 1.0
  27032.3826310              0.21726302465D-03
   4052.3871392              0.16838662199D-02
    922.32722710             0.87395616265D-02
    261.24070989             0.35239968808D-01
     85.354641351            0.11153519115
```

```
     31.035035245            0.25588953961
0 0 2 2 1.0
     12.260860728            0.39768730901
      4.9987076005           0.24627849430
0 0 1 0 1.0
      1.1703108158           1.0000000
0 0 1 0 1.0
      0.46474740994          1.0000000
0 0 1 0 1.0
      0.18504536357          1.0000000
0 2 4 4 1.0
     63.274954801            0.60685103418D-02
     14.627049379            0.41912575824D-01
      4.4501223456           0.16153841088
      1.5275799647           0.35706951311
0 2 1 0 1.0
      0.52935117943           .44794207502
0 2 1 0 1.0
      0.17478421270           .24446069663
0 3 1 0 1.0
      2.31400000             1.0000000
0 3 1 0 1.0
      0.64500000             1.0000000
0 4 1 0 1.0
      1.42800000             1.0000000
13  10
0 0 7 2.0 1.0
```

```
  37792.5507720       0.00057047888709
  5668.06821650       0.00440930165380
  1289.85828410       0.02263096741100
  364.865960280       0.08802564429500
  118.576315150       0.25223701612000
  42.0248676050       0.45960547169000
  15.4995016290       0.33277886014000
0 0 3 2.0 1.0
  75.2080265980       0.01925056019000
  23.0314089720       0.08790674395200
  3.63487976490      -0.34246704535000
0 0 2 2.0 1.0
  1.95000627000       1.01062661000000
  0.96103395000       0.38071016000000
0 0 1 0.0 1.0
  0.39881874000       1.00000000000000
0 0 1 0.0 1.0
  0.14940937000       1.00000000000000
0 2 5 6.0 1.0
  452.523031920       0.00231108124660
  107.081950490       0.01856864182300
  34.1310212550       0.08721623703500
  12.5870374280       0.26902101523000
  4.98119197040       0.52128324272000
0 2 1 1.0 1.0
  1.93791887000       1.00000000000000
0 2 1 0.0 1.0
```

```
  0.72047185000      1.00000000000000
0 2 1 0.0 1.0
  0.18328583000      1.00000000000000
0 3 1 0.0 1.0
  0.57798580000      1.00000000000000
8 8
0 0 6 2.0 1.0
  27032.382631      0.00021726302465
  4052.3871392      0.00168386621990
  922.32722710      0.00873956162650
  261.24070989      0.03523996880800
  85.354641351      0.11153519115000
  31.035035245      0.25588953961000
0 0 2 2.0 1.0
  12.260860728      0.39768730901000
  4.9987076005      0.24627849430000
0 0 1 0.0 1.0
  1.0987136000      1.00000000000000
0 0 1 0.0 1.0
  0.3565870100      1.00000000000000
0 2 4 4.0 1.0
  63.274954801      0.0060685103418
  14.627049379      0.0419125758240
  4.4501223456      0.1615384108800
  1.5275799647      0.3570695131100
0 2 1 0.0 1.0
  0.5489735000      1.0000000000000
```

```
0 2 1 0.0 1.0
  0.1858671100       1.0000000000000
0 3 1 0.0 1.0
  0.4534621300       1.0000000000000
99 0
```

Calculations details

The GGA XC functional PBE was used in all the calculations. D3 correction was used for inclusion of the non-covalent interactions. A highly fine integration grid was employed, using the keyword XXLGRID. For the SCF procedure, a tolerance of 10^{-10} was selected. For the integration tolerances, the set of values *8 8 8 8 20* was used. Different SHRINK parameters, which control the Monkhorst-Pack and Gilat nets k-space sampling, were chosen based on each unit cell size and are listed in Table S1.

Table S1: SHRINK values used for the different system sizes selected. Number of unit cells refer to the original unit cell of the pristine graphene system. *While considerably smaller to the rest, values for the largest systems where chosen in order to have affordable calculations. The consistent band gap value, with respect to those from freestanding monolayers counterparts, backup this selection.

N. unit cells	1	3	4	8	4(Slab)	8(Slab)
SHRINK	64 128	48 96	48 96	48 96	20 40	3 6*

Surface formation energy

In order to select the amount of atomic layers to be considered in our surface model, a test on the convergence of the surface formation energy was performed.[6] This energy can be computed using the following expression:

$$\Delta E^{n}_{surf} = \frac{(E^{n} - nE_{bulk})}{2A}, \tag{1}$$

with E^n the energy of a slab with n-layers, E_{bulk} the energy of a single layer's worth of bulk material, and A the surface area in the considered slab unit cell (in our case, 19.573 Å^2). An important thing to take intro account is that depending on the unit cell type, stoichiometry and facet to be used only certain numbers of atomic layers can be considered. In our case, slabs with a thickness of $3n$ atomic layers keep the slab to be Al-terminated on both ends, so an actual layer of (0001) α-Al_2O_3 should consider 3 atomic layers. The E_{bulk} in Equation (1) has to follow such requirement. The unit cell of a single layer of (0001) α-Al_2O_3 contains 2 Al atom and 3 of O (i.e. one formula unit). In the bulk unit cell we have 2 formula units, which means that E_{bulk} in Equation (1) is one half of the energy obtained from the bulk calculation.

An analogous expression, considering $E_{bulk} = E^n - E^{n-1}$ is:

$$\Delta E^n_{surf} = \frac{E^n - n(E^n - E^{n-1})}{2A}, \tag{2}$$

where all the quantities involved come from slab calculations, avoiding possible errors than arise when using both slab and bulk ones, as in (1). Both expressions should individually converge for a thick enough slab model. For our (0001) α-Al_2O_3 surface model, we can observe that at 18 atomic layers we get convergence using both expressions (Figure S26), for which we have chosen this value for our study (Figure S27). The surface energy calculated is around 2.1 J/m^2, in agreement with reference.[7]

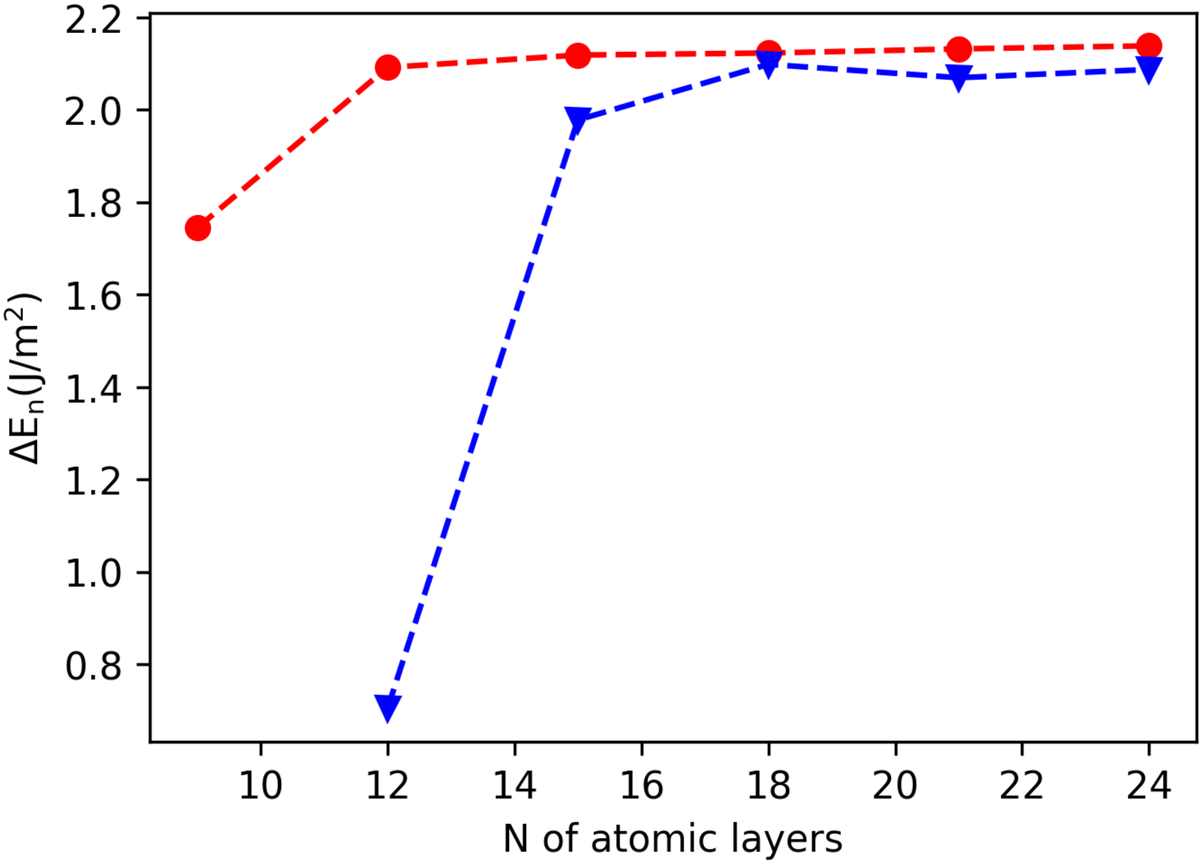

Figure S26: Surface formation energy for (0001) α-Al_2O_3, calculated both using Equation (1) (red circles) and Equation (2) (blue triangles).

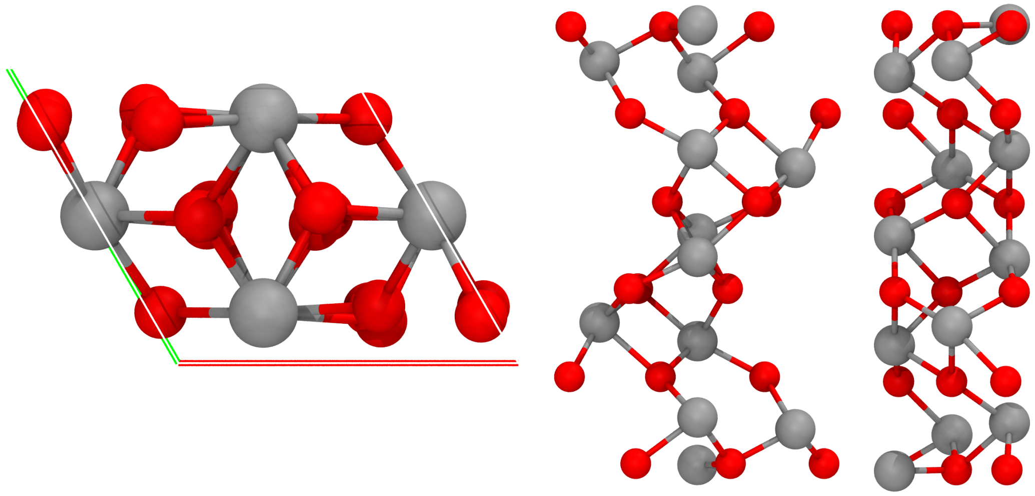

Figure S27: Unit cell of the slab model for Al-terminated (0001) α-Al_2O_3 with 18 atomic layers.

Electrostatic potential map for Al-terminated (0001) α-Al_2O_3.

The EP map calculated displays negative EP regions largely distributed, mainly on top of the Al atoms of the third and forth atomic layers. On top of the outer layer Al atoms a localized positive EP region observed. From this observation, we aligned the pristine and BCN freestanding system in such a way to favor the interaction between the outer Al atoms from our surface model and the negative EP regions from the monolayers. In order to avoid a large exploration of the PES, only one Monolayer@Surface proposal was considered, not testing alignments requiring rotation between systems and using a single unit cell of the Al-terminated (0001) α-Al_2O_3 slab.

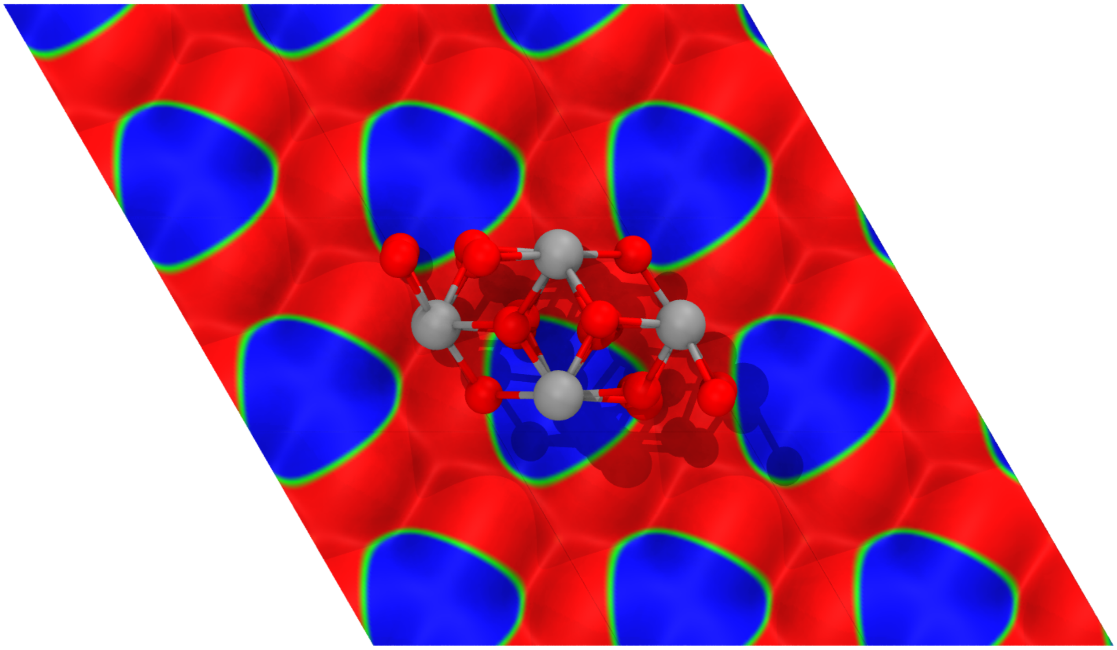

Figure S28: EP map on top of the slab model used, as described in the previous SI section. ρ_{cut} = 0.003, on the [-0.01, 0.01] EP range (red-green-blue color scale). A fragment of the system is shown on top of the ρ surface as reference.

Lattice mismatch between monolayers and Alumina surface

To measure the misalignment between the supported monolayers and the Alumina slab, we calculated the lattice mismatch for both a and b lattice parameters, as shown in Table S2. The percentage was calculated as

$$\frac{\Delta p}{p}\% = \frac{p_{layer} - p_{slab}}{p_{slab}} \times 100\%, \quad (3)$$

where p can be the a or b lattice parameters. The values employed were those of the optimized structures as obtained with the previously described computational methodology. In all cases we observe positive mismatch percentages. On each supported pair the monolayer was initially adjusted to the slab, imposing a compression over it.

Table S2: Lattice mismatch percentages between the monolayers and the Alumina slab, ordered in increasing amount of BN pairs in the monolayer.

Parameter / System	pG	ac2	ac4	ac1	alt1	zz1	i1	i2	pBN
a	1.73	2.40	2.42	3.29	3.26	3.32	3.42	3.34	3.52
b	1.73	2.40	2.42	3.29	3.28	2.31	3.42	3.34	3.52

Analysis of the interaction between H_2O and freestanding and Alumina-supported graphene

The calculation for H_2O@Alumina-supported graphene shows that the E_{int} is lower with respect to the freestanding case (-132.71 and -190.52, respectively, considering the most similar H_2O final geometries of each case). Unexpectedly, the H_2O-Monolayer distance on the supported system was lower (2.36 Å against 2.51 Å). A QTAIM analysis,[8] using TOPOND as included in CRYSTAL23[9] and which results can be seen in Table S3, also points that the stronger interaction should be found for the supported case (larger ρ at the bond critical

point). Interestingly, the ellipticity for the supported case is considerably larger, which indicates a more delocalized interaction .

Table S3: QTAIM descriptors for the interaction H_2O-graphene's C atom. ρ_{BCP} is the ρ value at the bond critical point, $\nabla\rho_{BCP}$ is the Laplacian of ρ at that same value, and ϵ is the ellipticity.

	Freestanding	Supported
ρ_{BCP}	0.008	0.011
$\nabla^2\rho_{BCP}$	0.025	0.030
ϵ	0.634	1.041

As a way to explain this observation we compared some of their quantities based on their electron densities (ρ). First we checked the ρ difference between the system and the promolecular ρ (i.e. the electron density which results from summing the electron densities of the conforming isolated atoms), here indicated as $\Delta\rho$. Figure S29 displays maps for this difference for each system. We observe that, while small, there is a difference in how ρ is distributed above bonded C atoms of graphene. with a reduction on the extension of the charge depletion area, which are areas with increased ρ values. This reduction of the electron-rich region can both allow the closer approach of the H_2O molecule as well as decrease the strength of the interaction with its electron-deficient H atoms.

Furthermore, we calculated the 3-dimensional deformation densities of the substrates interacting with H_2O, $\Delta\rho_{int}$, calculated as

$$\Delta\rho_{int} = \rho_{H_2O-Substrate} - (\rho_{H_2O} + \rho_{Substrate}), \tag{4}$$

with $\rho_{H_2O-Substrate}$ the electron density of the optimized H_2O-Substrate complex. ρ_{H_2O} and $\rho_{Substrate}$ are obtained from single point calculations at the optimized geometry in the complex. To estimate this difference the 3-dimensional ρ scalar field was calculated using CRYSTAL (module properties, keyword ECH3), using the same sampling grind in all cases, generating files with *.cube* format. Given that the grids are compatible (i.e. same number of points and same unit cell), the difference can be easily computed as the difference in value

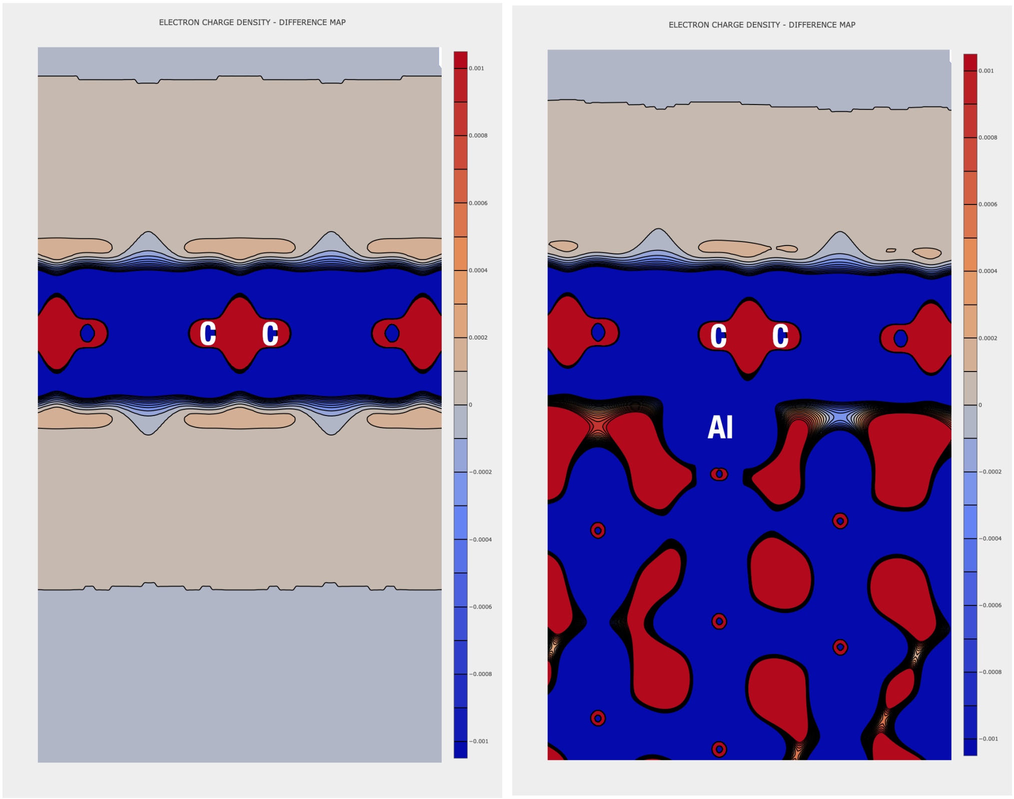

(a) free-standing Graphene (b) Alumina-supported Graphene

Figure S29: $\Delta\rho$ maps for free-standing (a) and Alumina-supported (b) Graphene, on the plane C-C-X (X being a point above the first C atom and along the normal vector of the graphene plane). Labels indicate the location of atoms. Plots generated using CRYSPLOT.[10]

at each grid point, which results in a new *.cube* file. A simple C++ program to perform this difference is available at `https://github.com/molecular-mar/cube_diff`.

The deformation in the electron density, as displayed in Figure S30, due to the presence of the H_2O molecule is larger in the case of the supported systems, covering a larger amount of C atoms (3 instead of 1) at the interaction site. This larger deformation has a positive sign, which indicates a positive deformation (larger ρ values in the complex with respect to its individual components). Such change is consistent with the QTAIM results (larger ellipticity and larger ρ_{BCP}), and can be consider as evidence of a larger destabilizing effect on the graphene layer, for which the estimated interaction energy is lower.

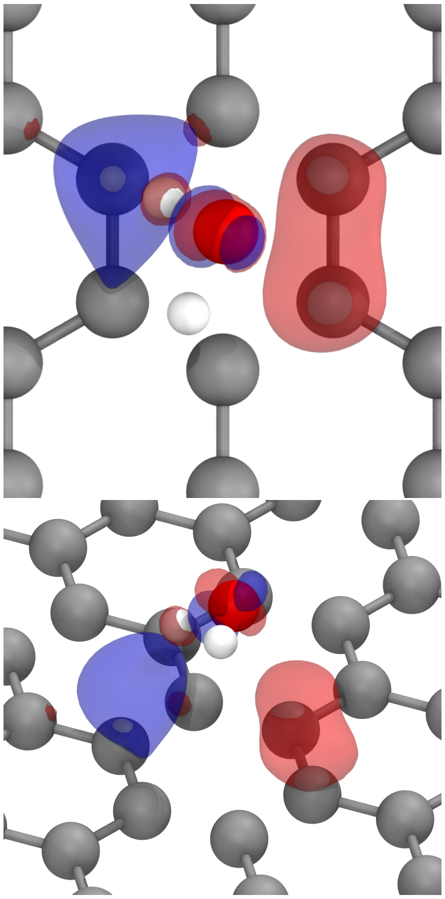

(a) freestanding Graphene

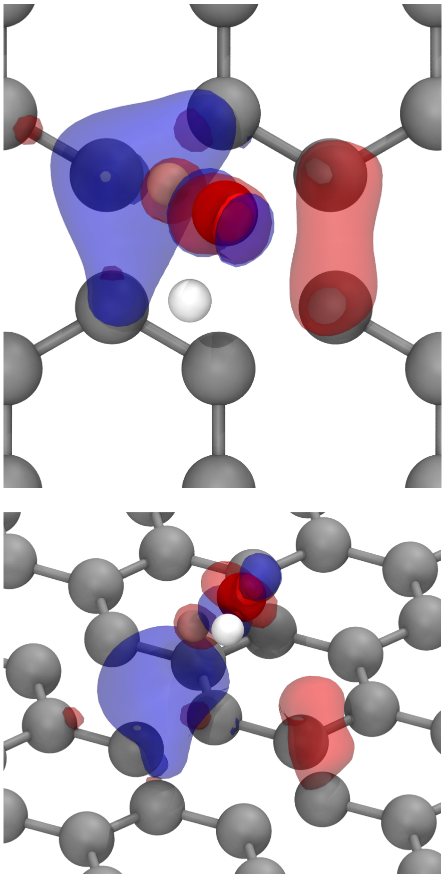

(b) Alumina-supported Graphene

Figure S30: Deformation density for freestanding (a) and Alumina-supported (b) Graphene interacting with a H_2O molecule. Red zones indicate a negative deformation (lower ρ value in comparison to the individual components), and blue zones indicate a positive deformation.

References

(1) Daga, L. E.; Civalleri, B.; Maschio, L. Gaussian Basis Sets for Crystalline Solids: All-Purpose Basis Set Libraries vs System-Specific Optimizations. *Journal of Chemical Theory and Computation* **2020**,

(2) Vilela Oliveira, D.; Laun, J.; Peintinger, M. F.; Bredow, T. BSSE-correction scheme for consistent gaussian basis sets of double- and triple-zeta valence with polarization quality for solid-state calculations. *Journal of Computational Chemistry* **2019**, *40*, 2364–2376.

(3) Schuchardt, K. L.; Didier, B. T.; Elsethagen, T.; Sun, L.; Gurumoorthi, V.; Chase, J.; Li, J.; Windus, T. L. Basis Set Exchange: A Community Database for Computational Sciences. *J. Chem. Inf. Model.* **2007**, *47*, 1045–1052.

(4) Feller, D. The role of databases in support of computational chemistry calculations. *J. Comput. Chem.* **1996**, *17*, 1571–1586.

(5) Pritchard, B. P.; Altarawy, D.; Didier, B.; Gibsom, T. D.; Windus, T. L. A New Basis Set Exchange: An Open, Up-to-date Resource for the Molecular Sciences Community. *J. Chem. Inf. Model.* **2019**, *59*, 4814–4820.

(6) Dovesi, R.; Civalleri, B.; Roetti, C.; Saunders, V. R.; Orlando, R. *Ab Initio Quantum Simulation in Solid State Chemistry*; 2005; Vol. 21; pp 1–125.

(7) Tepesch, P. D.; Quong, A. A. First-Principles Calculations of α-Alumina (0001) Surfaces Energies with and without Hydrogen. *physica status solidi (b)* **2000**, *217*, 377–387.

(8) Bader, R. F. W. *Atoms in Molecules: A Quantum Theory*; Oxford University Press: Oxford, 1990.

(9) Cossard, A.; Desmarais, J. K.; Casassa, S.; Gatti, C.; Erba, A. Charge Density Analysis of Actinide Compounds from the Quantum Theory of Atoms in Molecules and Crystals. *The Journal of Physical Chemistry Letters* **2021**, *12*, 1862–1868.

(10) Beata, G.; Perego, G.; Civalleri, B. CRYSPLOT: A new tool to visualize physical and chemical properties of molecules, polymers, surfaces, and crystalline solids. *Journal of Computational Chemistry* **2019**, *40*, 2329–2338.
